# Geomagnetic reversal rates following Palaeozoic superchrons have a fast restart mechanism

**DOI:** 10.1038/ncomms12507

**Published:** 2016-08-30

**Authors:** Mark W. Hounslow

**Affiliations:** 1 Lancaster Environment Centre, Lancaster University, Bailrigg, Lancaster LA1 4YB, UK

## Abstract

Long intervals of single geomagnetic polarity (superchrons) reflect geodynamo processes, driven by core–mantle boundary interactions; however, it is not clear what initiates the start and end of superchrons, other than superchrons probably reflect lower heat flow across the core–mantle boundary compared with adjacent intervals. Here geomagnetic polarity timescales, with confidence intervals, are constructed before and following the reverse polarity Kiaman (Carboniferous–Permian) and Moyero (Ordovician) superchrons, providing a window into the geodynamo processes. Similar to the Cretaceous, asymmetry in reversal rates is seen in the Palaeozoic superchrons, but the higher reversal rates imply higher heatflow thresholds for entering the superchron state. Similar to the Cretaceous superchron, unusually long-duration chrons characterize the ∼10 Myr interval adjacent to the superchrons, indicating a transitional reversing state to the superchrons. This may relate to a weak pattern in the clustering of chron durations superimposed on the dominant random arrangement of chron durations.

## Abstract (web summary)

The mechanism behind the initiation and termination of superchrons remains unclear. Here, the author provides insight into Palaeozoic superchrons, showing that the 10 million year interal adjacent to each superchron is characterized by asymmetry in reversal rates and unusually long duration chrons.

## Graphical abstract

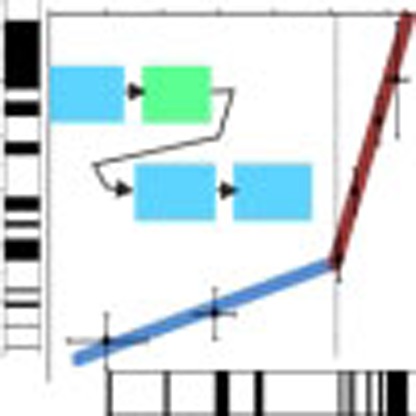

Non-reversing (superchrons) and reversing geodynamo states punctuate the Phanerozoic and Proterozoic^1^, with reverse polarity superchrons during the early-mid-Ordovician (Moyero superchron) and during the late Carboniferous–mid Permian (Kiaman superchron) and normal polarity superchrons during the late Cretaceous (CNPS) and Proterozoic. The superchron transitions are thought to reflect a threshold between reversing and non-reversing states, with reducing heatflow (at superchrons' start) or increasing heatflow (at superchrons' end), across the core–mantle boundary (CMB)^2^. The heat flow changes likely respond mainly to mantle convention, since convective turnover time in the core is much shorter than that in the mantle. However, geodynamo models driven by geologically constrained mantle convention models^3^,^4^ highlight the different model heatflow constraints required to simulate the CNPS compared with the Kiaman and Moyero superchrons. Plate tectonic-driven models of the mantle suggest an increased heatflux during the CNPS, not reduced heatflux, and require that the lower mantle may be de-coupled or insulated from the main mantle flow^5^. This conundrum has resulted in the ‘superplume' hypothesis^6^, in which the thermochemical piles in the lower mantle are inferred to expand upwards and later shrink on an ∼200 Ma repeat time controlling the superchron cycles. More rapidly reducing CMB heatflow is inferred during the collapse phase of the ‘superplume'. Support for this hypothesis is the asymmetry in the polarity-reversal rates on either side of the CNPS, with lower reversal reversal rates following the end of the CNPS. It is not clear whether there is support for this ‘superplume' model in the Palaeozoic superchrons, since reversal rates are an open discussion because of a lack of robust data. In the Mesozoic geomagnetic polarity timescale (GPTS), recovery from the CNPS starts with two long-duration chrons (C33r and C33n) of some 4–5.5 Myr duration. This is followed by either stationary reversal behaviour^7^, or a slow increase in reversal rates, over some 50 Myr before reversal rates stabilize to what they have been over the last 30 Myr (refs 7, 8). Slowly increasing reversal rates are also inferred following the Kiaman superchron, during the mid and late Permian into the Triassic^4^,^9^. Crucially, the characteristics of the palaeomagnetic properties (rates of reversals, secular variation and field intensity) around the superchron start and end transitions hold clues to interpreting the geodynamo response^10^. Initiation of all superchrons is normally inferred to be pre-empted by a more rapid drop in reversal rates, evidenced by longer chrons immediately pre-superchron, as exemplified during the early Cretaceous^10^.

This work examines the 0–15 Myrs before and following the end of the Palaeozoic superchrons by providing a quantitatively derived polarity timescale as a proxy for the core–mantle behaviour driving the geodynamo. Comparisons of the reversal structure adjacent to the Phanerozoic superchrons provide clues to differences in the ending and restart conditions of the reversing geodynamo.

## Results

### Construction of geomagnetic polarity timescales

**Figure 1:**
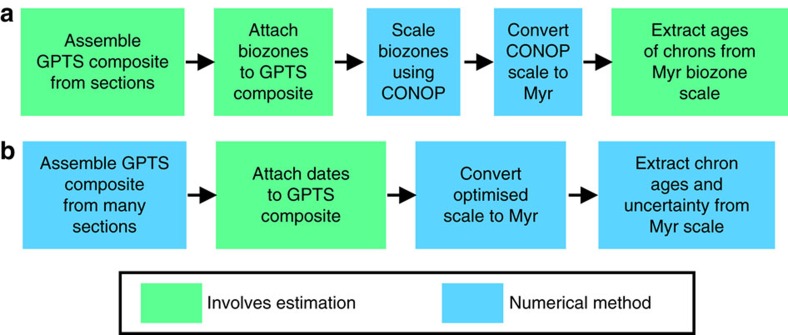
Types of procedures used in polarity timescale constructions. (**a**) Procedures in geomagnetic polarity timescale construction for the Permian, Carboniferous and Ordovician as used in the 2012 timescale^16^,^17^ versus (**b**) the optimization method used here. The main additional procedures used in the 2012 polarity timescale in (**a**), but not used in the optimization method here, are the transfer through the CONOP-scaled biozones, the need to estimate polarity boundary position in the scaled biozones and the absence of chron uncertainty assessment.

**Figure 2:**
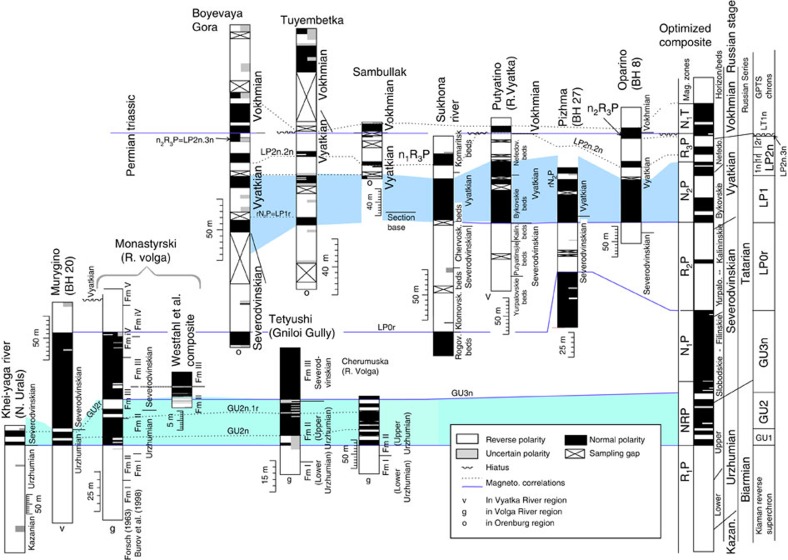
Mid and late Permian magnetostratigraphy from Russia. Summary of section data from successions west of the Urals, with additional supporting stratigraphic details in Supplementary Table 1. Boyevaya Gora, Tuyembetka and Sambullak sections are near Orenburg^37^. Murygino, Tetyushi, Cherumuska, Putyatino, Pizhma and Oparino sections are near the Kama, Volga and Vyatka Rivers (SW Tataria, Kazan region), some ∼700 km NE of the Orenburg area^22^. Monastyrski (Volga River) section is from the Kazan region^22^,^61^. Sukhona River section is from NE Russian, ∼600 km North of Kazan^70^. Khei-Yaga River section is from the Northern Urals^64^. Each section has a thickness scale in metres. Composite magnetozones are labelled with the Russian naming convention^21^,^22^ and the inferred correlative chrons. BH, borehole number.

**Figure 3:**
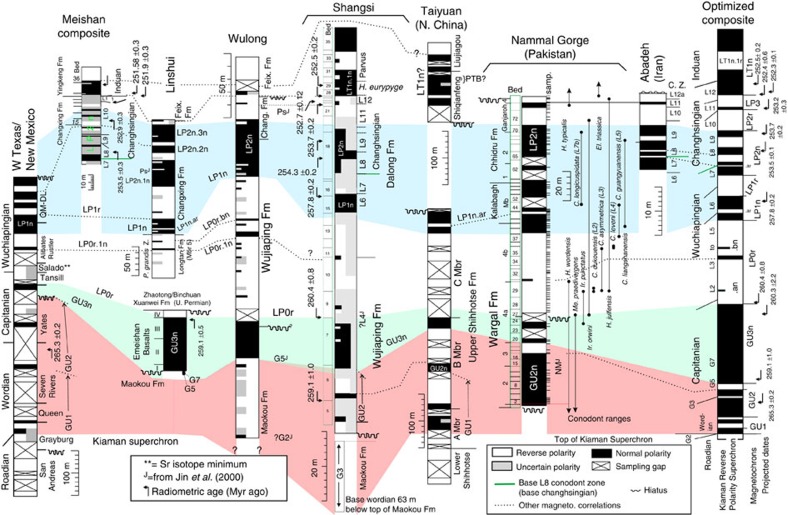
Mid and late Permian magnetostratigraphy from other marine and non-marine sections. Additional supporting stratigraphic details in Supplementary Table 1. West Texas and New Mexico geomagnetic polarity^26^ show radiometric dates related to the lithostratigraphy via sequence correlation. The Meishan magnetic polarity is a composite^29^,^30^,^31^. The Linshui section magnetostratigraphy^19^ has a revised biostratigraphy. Emeishan Basalt magnetostratigraphy is a section composite^32^,^34^,^35^. The biostratigraphy of the Wulong section^13^ is not adequately documented, but the magnetostratigraphy^19^,^25^ appears to range into the lower Capitanian. The Shangsi magnetostratigraphy is a composite section^65^,^66^,^67^. The magnetostratigraphy from the lowest part of the Taiyuan (non-marine) section defines the end of the Kiaman Superchron^27^. The Nammal Gorge section magnetostratigraphy^18^ has a correlated conodont biostratigraphy based on nearby sections. The Abadeh magnetostratigraphy^24^ and its associated conodont biostratigraphy primarily allows conodont zonations to be tied to the GPTS in the Changhsingian. Fusulinids: Ps=*Palaeofusulina* spp., Nm=*Neoschwagerina margaritae*. Conodont zones: G2=*J. asserata* (base Wordian), G3=*J. postserrata* (base Capitianian), G5=*J. altudaensis* (mid Capitanian) and G7=*J. xuanhanensis* (upper Capitanian). L1 to L12 are the standard Lopingian conodont zones^11^. Ammonoid zones: T–S=*Tapashanites*–*Shevyrevites* assemblage zone; P–P=*Pseudotirolites*–*Pleuronodoceras* assemblage Zone.

**Figure 4:**
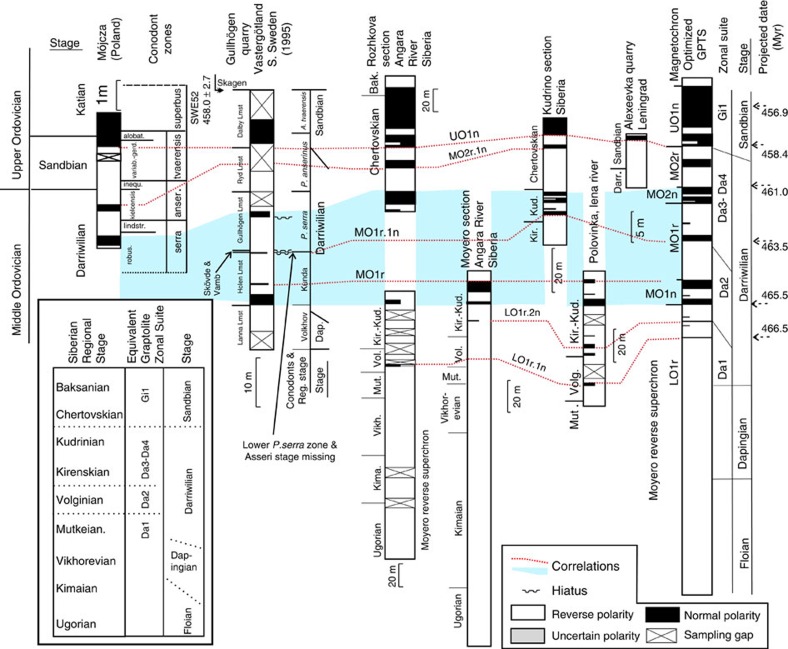
Summary of Middle and Upper Ordovician magnetostratigraphic data. Polish and Swedish data from the Mójcza^47^ and Gullhögen sections^48^. The Polovinka section data merge data from two sections, with ‘profile-1' representing the member-7 interval^68^. Siberian data from the Rozhkova^44^, Moyero^14^,^39^, Kudrino and Alexeevka sections^43^. Shown on the left are proposed relationships of the Siberian regional stages to graptolite zones and international stages^46^.

A robust construction of the polarity-reversal structure following the Kiaman and Moyero superchrons has not been made. Proposed end-Kiaman GPTS has both long chrons of 2–3.5 Myr duration^11^,^12^ or numerous briefer chrons^13^. The data sets immediately before the Kiaman and Moyero superchrons (mid-Carboniferous and late-Cambrian–early Ordovician respectively) are less extensive^14^,^15^. In the 2012 GPTS timescale for the Permian, Carboniferous and Ordovician, while the faunal ranges are scaled and converted into a composite position-scale using CONOP^16^,^17^, the polarity chrons are qualitatively attached to the biozonal scale, at the last stage, after the biozones are scaled to Myrs (Fig. 1). These procedures lose the information in the relative duration of chrons embedded in the section data, but introduce additional guesswork, since the chron boundary positions in the scaled biozones need to be estimated in ways that are not defined. The later step also has considerable uncertainty, since relationships between chron boundaries and relevant biozonal boundaries are often not well defined^17^ (see uncertainty in biozone placement in Figs 2, 3, 4). The 2012 mid-late Permian GPTS^12^ is also based on only limited primary data sets from the Nammal Gorge, Wulong and Linshi sections^18^,^19^. The mid-late Ordovician GPTS in the 2012 timescale^16^ is based on the small composite figure in ref. 14, with primarily the old British Ordovician regional stages for reference. The Carboniferous and late-Cambrian–early Ordovician GPTS in the 2012 timescale have similar limitations.

Here a new more inclusive numerical approach is used by attaching the radiometric dates directly to a composite geomagnetic polarity scale, which uses multiple section data. Critically, this removes the transfer through a biozone scale to get at the estimated radiometric ages for the chrons (Fig. 1). The GPTS produced uses a numerical optimization procedure, which generates a statistical composite of the geomagnetic polarity, initially in a scale of composite section height. The numerical method finds a solution that minimizes the misfits between the final composite and the section data, subject to chosen transformations that modify the height scales of the sections. From the final model composite, several statistical measures are determined. First, *D*_*s*_ that assesses the proportion of relative misfits of the overall model; second, *σ*_*T*_ (in Myr), the s.d. of the transformed chron positions in the sections (about the position of the chron in the composite); third, *D*_*j*_ that measures the relative misfit of the magnetozone data in each section with respect to the final composite. These allow comparative assessment of the data consistency and the chosen transformations. Importantly, the method also produces confidence intervals on the chron ages. This new quantitative approach allows a more comprehensive assessment of the GPTS interval, since, most importantly, it directly uses the proxy for duration embedded in magnetozone-relative thicknesses in numerous sections.

In the new Palaeozoic polarity composites, the magnetochrons have been labelled in groups, corresponding to polarity dominance with a series pre-fix (for example, MI1 to MI7 for the Mississippian in the Carboniferous, Supplementary Fig. 1; GU1 to GU3n, LP1 to LP3, for the Guadalupian (Middle) and Lopingian (Upper Permian; Figs 2 and 3). The Permian Russian regional labelling scheme is also shown in Fig. 2. The late Cambrian–Ordovician magnetochrons are labelled mC (informal mid Cambrian), FU (Furongian), and LO, MO and UO (for Lower, Middle and Upper Ordovician, respectively, Fig. 4 and Supplementary Fig. 2). Data used in this compilation are all based on modern palaeomagnetic cleaning techniques, with good sampling density coverage, and data used are displayed with respect to section height, giving magnetostratigraphic data-quality values^20^ typically in excess of 7. The compositing procedure used here needs the largest possible subset of the stratigraphically most detailed and most reliable polarity data, with high sampling density, reasonable biostratigraphy and the largest stratigraphic coverage. Filtering the data set to that with the highest quality critera^20^ would result in a lower resolution and less detailed polarity timescale. Some data interpretations have been slightly modified from original publications to maintain a consistency in polarity boundary interpretation between data sets.

### Russian Permian data

In Russian sections, the end of the Kiaman superchron is in the upper Urzhumian^21^,^22^. Multiple sections, borehole cores and studies through the Kazanian and Lower Urzhumian have failed to detect any normal polarity intervals below the Russian magnetozone NRP; therefore, the end of the Kiaman Superchron is clearly expressed in these extensive data^22^. The NRP Russian polarity interval shows two major reverse polarity intervals, the upper one of which is subdivided by a normal polarity submagnetozone. The structure of the lower reverse magnetozone in the NRP polarity interval is less clear and appears to be best represented by the Cherumuska section (type section of the Urzhumian), which is particularly thick. The uppermost normal polarity parts of magnetozone R_3_P (that is, n_1_R_3_P and n_2_R_3_P) are missing from some sections, but are clearly present in the Oparino core and Boyevaya Gora section (and other sections^22^ not illustrated in Fig. 2).

Russian workers split the normal magnetozone sometimes seen straddling the Vyatkian–Vokhmian boundary into n_2_R_3_P and N_1_T. The Vyatkian part is below a major increase in susceptibility and remanence intensity (because of a regional magnetite abundance increase) and a Vokhmian part above^22^. The latest Permian magnetozones, LP2n to LP3, are variably removed by erosion at the base of the Vokhmian, suggesting that magnetozone n_2_R_3_P is the equivalent of LP2n.3n (Figs 2 and 3).

Marine sections display more detail in magnetozones over the Lopingian part of the timescale (Fig. 3) than the Russian non-marine sections, whereas the Russian platform sections show more detail in the Guadalupian (GU1 to GU3n intervals; Fig. 2). This is probably because of the absence of the mid and upper Changhsingian in many Russian sections^23^.

### Permian marine sections and Chinese sections

The middle and upper Permian magnetostratigraphy from marine sections and Chinese sections display the greatest detail and resolution in the Wuchiapingian and Changhsingian (that is, Abadeh, Linshui and Wulong sections^19^,^24^,^25^), whereas the Capitanian and Wordian magnetostratigraphy is less well defined (Fig. 3). The end of the Kiaman Superchron is shown in the West Texas/New Mexico data^26^, the Taiyuan section^27^ (Fig. 3) and in data from Japan^28^. The magnetochron GU1n is the ‘Word-N' magnetozone of ref. 26, which in the sections in the American south west appears to place it in the early Wordian.

Magnetostratigraphic data from Permian limestones in Japan^28^ show GU3n in the mid Capitanian overlain by reverse polarity in the early Wuchiapingian, where their section-8 has an additional normal polarity magnetozone near the start of the Wuchiapingian, which is not clearly shown in other data. Their magnetostratigraphy through the Wordian contains both reverse and normal polarity intervals, with the earliest normal polarity magnetozone in the *Neoschwagerina craticulifera* fusilinid zone (their section 2) of the early Wordian, which may be the base of GU1n or GU1r.1n. The GU3n chron is the ‘Capitan-N' magnetozone of ref. 26. The fragmentary (but well-dated) nature of the sections in Japan does not allow them to be used in the composite GPTS construction.

In spite of three studies^29^,^30^,^31^ on the Changhsingian at Meishan (the Changhsingian GSSP), the agreement on the magnetic polarity is poor. The only consistency between these studies suggests a lower normal polarity interval (the LP2n.1n?) and mixed polarity in the younger part of the Changhsingian (LP2n.3n-LP3r polarity interval).

The age of the base of the Linshui section (Member 5 of the Lungtan Fm) is based on regional correlation of brachiopod assemblages, suggesting a late Wuchiapingian age, supported by the presence of the conodont *Clarkina liangshanensis* from the basal beds of the Lungtan Fm, 300 m below the measured magnetostratigraphy (personal communication, Shu-zhong Shen, 2010).

The Ebian county magnetostratigraphy through the Emeishan Basalts^32^ along with the Guadalupian conodonts *Jinogondolella altudaensis* (zone G5, Fig. 3) and *J. xuanhanensis* (zone G7), from the underlying few metres of the Maokou Fm^33^ (and radiometric dates), suggest a mid to late Capitanian age for the Emeishan Basalts. The Emeishan Basalt units continue into the overlying reverse polarity basal Wuchiapingian^32^,^34^,^35^.

The Nammal Gorge magnetostratigraphic data^18^ have little supporting published biostratigraphic data, but conodont ranges (Fig. 3) can be related to the magnetostratigraphic data using nearby sections^36^. These are correlated onto the magnetostratigraphic section using published logs and other stratigraphic details (see Supplementary Table 1). The brief normal chron LP0r.1n (P3 magnetozone of ref. 26) is clearly shown in the Wulong and Linshui sections in China and in the Rustler Fm in New Mexico. The base of LP1n is clearly identified in all sections that cover this late Wuchiapingian interval (Fig. 3). The three reverse magnetochrons in the interval LP1n–LP2n.2n shown in most sections seem to vary greatly in thickness (like the LP1r in the Russian sections from the Orenburg area^37^ (Fig. 2)).

**Figure 5:**
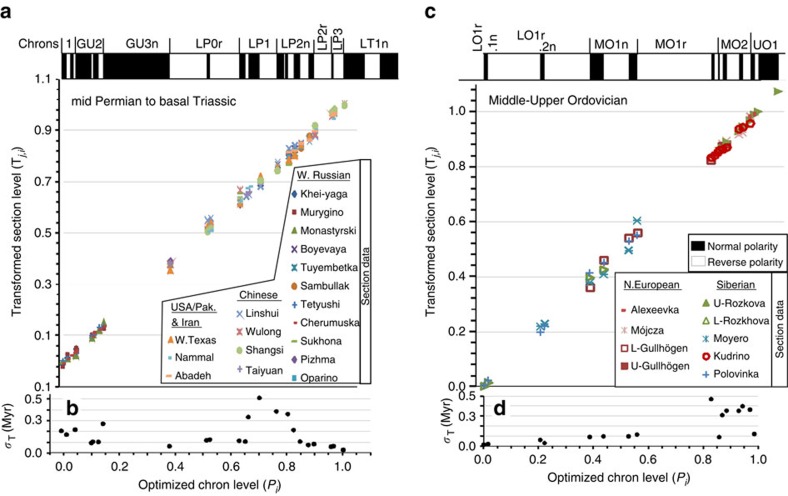
Optimized reversal stratigraphy for the Permian and Middle-Upper Ordovician. (**a**,**c**) The base of the transformed corresponding magnetozone from each section is shown on the *y* axis, along with the median chron level (**P**_*i*_) on the *x* axis and the resulting reversal stratigraphy in the top panel. (**b**,**d**) *σ*_*T*_—the s.d. of the data for chron **T**_*i*_ values displayed on the *y* axis. The s.d. of **T**_*i*_ is scaled to Myr using the chron durations derived from Figs 6b and 7b (see Supplementary Tables 5 and 7), which is why the scatter in **T**_*j,i*_ values on the *y* axis (in **a**,**c**) does not correspond visually to the magnitude of *σ*_*T*_ in Myr in (**b**,**d**). Carboniferous (Mississippian) and late Cambrian–Lower Ordovician optimization data shown in Supplementary Fig. 4.

**Figure 6:**
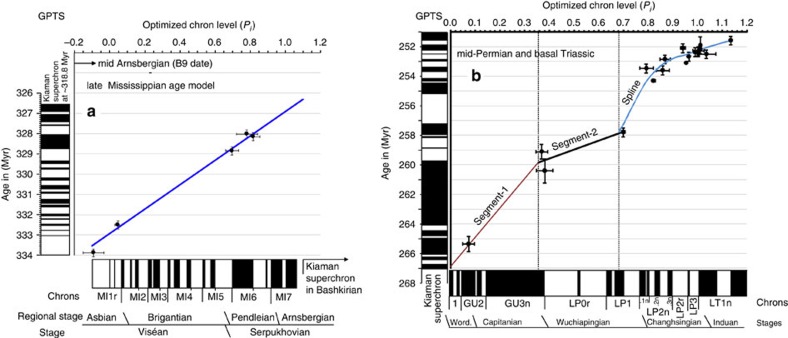
Age models for the Permian and Mississippian. Optimized reversal stratigraphy (on *x* axis) from Fig. 5a and Supplementary Fig. 4, and the final GPTS on the *y* axis. 2*σ* error bars on age control points (in Myr) and stratigraphic uncertainty (optimized scale units on the *x* axis) in placing age control point on magnetostratigraphy (see Supplementary Tables 2 and 3). Boundaries between timescale segments indicated by vertical dashed lines. The regression relationships between the optimized chron levels and age are shown for the three Permian segments and the one Carboniferous segment.

The optimized composite (Figs 5a and 6b) differs considerably from previous attempts^13^,^26^, primarily by not using the Meishan section for scaling, and the smaller number of brief normal polarity intervals in the lower Wuchiapingian (Fig. 3). The most recent inclusive attempt^26^ is somewhat similar in the Wuchiapingian–Changhsingian, but differs greatly in relative thickness of magnetozones, especially for the Capitanian–Wordian. The most complete and well-documented transition into chron LT1n (which includes the Changhsingian–Induan boundary) is at the Shangsi section, where the chrons can be tied to a succession of conodont zones^38^.

### Carboniferous geomagnetic polarity data sources

The most detailed magnetic polarity data are available for the Mississipian (mid-Carboniferous) from North America^15^. Rather, more fragmentary magnetic polarity data occur in the early Pennsylvanian, which however define the base of the Kiaman superchron (Supplementary Fig. 1) in the late Bashkirian (upper part of European Yeadonian regional stage^15^). The base Kiaman is approximately at *ca.* 318.8 Myr, derived from an array of radiometric dates through the Bashkirian and Moscovian from Russian sections, which can be related to the European stages using biostratigraphy^17^. A gap of some *ca.* 6 Myrs occurs between the late Mississippian and early Pennsylvanian magnetic polarity data sets; therefore, the magnetic polarity data are incomplete before the start of the Kiaman superchron^15^.

### Late Cambrian–Ordovician geomagnetic polarity data sources

Palaeomagnetic polarity data for the Ordovician define the base of the Moyero superchron in the Tremadocian^14^. Magnetic polarity data from the underlying late Cambrian are known from Siberia^39^,^40^, Australia^41^ and China^42^ (Supplementary Fig. 2). The key Siberia data set (from the Kulyumbe section) can be related to the well-dated (using conodonts) Australian data set by a set of carbon isotope-negative and -positive excursions in the late Cambrian^40^. Attempts at extending the GPTS further into the mid Cambrian using the Kulyumbe section data are hindered by an interval of low-quality data in the Orakta Fm^40^.

The upper boundary of the Moyero superchron is in the early part of the Middle Ordovician^43^. No detailed magnetic polarity data are available for the mid Katian or younger in the late Ordovician. The mid and late Ordovician magnetic polarities from Siberia (Fig. 4) are tied to the Siberian regional stages^43^,^44^. However, these stages are problematic to correlate to the international Ordovician stages, since they are largely based on endemic shelly faunas^45^,^46^. In contrast, data from the Mójcza^47^ and the Gullhögen sections^48^ have a well-defined conodont-based biostratigraphy.

In Siberia, early Baksanian strata contain the graptolite *Oepikograptus bekkeri*^46^, which has been used as an analogy for the cosmopolitan early Sandbian, *Nemagraptus gracilis* Zone. (ref. 45) correlates the base of the Chertovskian to the base of the Sandbian, using co-occurring *N. gracilis*, and the regional equivalence of shelly faunas. This is similar to the proposed magnetostratigraphic correlation to the Gullhögen section. Not used in the composite construction are data from the Kulyumbe and Stolbovaya sections^43^, which cover the mid part of the Kirenskian–Kudrian interval and indicate uninterrupted reverse polarity (that is, chron MO1r; Fig. 4).

The base of sub-magnetochron LO1r.1n is used as the end of the Moyero superchron, one of two brief submagnetozones in the Siberian Volginian and lower Kirenskian stages in the lower Rozhkova, Polovinka and Moyero sections. Normal magnetochron MO1n.1n (Fig. 4), seen within the Kirenskian–Kudrenian regional stages, is the equivalent magnetozone to that seen in the lowest part of the Holen Limestone in Sweden, which is dated to the *Lenodus variabilis* conodont zone^49^. This is correlated with the Da1–Da2 (Darriwillian) zonal boundary^16^. The MO1r interval is reverse polarity-dominated, with only the Gullhögen section spanning this entire interval. The chron MO2n is best represented in the Siberian Kudrino section, but inferred to be present in the upper Rozhkova and Gullhögen sections. The high *σ*_T_ values (Fig. 5d) indicate that there is much variability in magnetozone-relative thicknesses in the MO1r.1n to UO1n.1n interval. The Darrawilian–Sandbian boundary is well defined in the Mójcza and Gullhögen sections within chron MO2r.2r (Fig. 4).

It has been suggested that the upper boundary of the Moyero superchron is at the base of MO1n; however, there is evidence for at least two brief normal chrons below this that are validated by single-sample data from two sections^43^. Other single-sample intervals are not validated; therefore, the assumption used here is that the end of the Moyero Superchron is the base of LO1r.1n.

### Radiometric age constraints

The Permian GPTS uses 20 U-Pb dates that can be directly related to the magnetostratigraphy (Fig. 6b), either within the sections or in sections that can be reliably tied to the sections with the magnetostratigraphy (Supplementary Table 2). Radiometric dates best constrain the Permian GPTS in the Changhsingian, and are sparse during the Wuchiapingian, Capitanian and Wordian (Fig. 6b). The composite Shangsi section data have a large number of associated U-Pb dates, making this section important for date-constraining the Lopingian magnetostratigraphy. The U-Pb radiometric ages from the Meishan section, using the EARTHTIME tracer calibration^50^, are offset (ranging from −0.087 to −0.157%) from the pre-EARTHTIME ages^38^ by some −0.126% on average (for beds 22, 25 and 28). This may bias the age calibration of the GPTS in the late Changhsingian; however, such offsets may not be systematic^51^, compared with older pre- EARTHTIME calibrations, so the most recently published U-Pb dates are used (Supplementary Table 2).

The Permian age model indicates the good definition of the chron ages between 251 and 255 Myrs (Fig. 6b and Supplementary Fig. 3). However, the small number of radiometric dates available for the 257 to 266 Myrs interval produce larger 95% confidence intervals for chron ages older than ∼259 Myrs, which are some two to three times larger than those in the Changhsingian (Supplementary Table 5). The single Wordian radiometric date (Supplementary Table 2) has the biggest impact on the age model in the Guadalupian (Fig. 6b).

The Carboniferous age model has five radiometric dates (Supplementary Table 3), which constrain a linear relationship between the optimized scale and age (Fig. 6a). A sixth radiometric date from the mid Arnsburgian (Supplementary Table 3, date B9), if projected into this relationship (Fig. 6a), suggests that the magnetic polarity data ends in the earliest part of the Arnsburgian (late Serpukovian^17^). Carboniferous optimization and confidence interval data are shown in Supplementary Figs 4a and 5.

**Figure 7:**
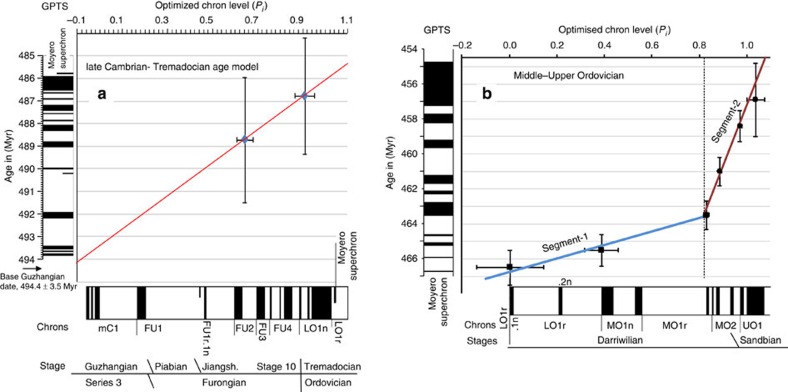
Linear age models for the mid Cambrian to Upper Ordovician optimized reversal stratigraphy. 2*σ* error bars on age control points (in Myr) and stratigraphic uncertainty (optimized scale units on *x* axis) in placing age control point on magnetostratigraphy (see Supplementary Table 4). Section polarity data in a) for mid Cambrian Lower Ordovician (Tremadocian) interval in Supplementary Fig. 2. Boundaries between timescale segments indicated by vertical dashed line in **b**.

In the mid–late Ordovician data, the only directly section-related radiometric date is from the Kinnekulle-K bentonite (^40^Ar/^39^Ar date of 458.0±2.7 Myr, recalculated^16^) at the top of the Dalby Limestone (Fig. 4). However, this part of the section has no magnetostratigraphy; therefore, its position with respect to the magnetostratigraphy cannot easily be inferred, hence is not used in the final data set to scale the polarity (Supplementary Table 4). Instead, CONOP-based age estimates of biostratigraphic boundaries (Table 20.1 in ref. 16) are used. The only radiometric date used is a ^206^Pb/^238^U date (456.9±2.1 Myr) from strata in the upper part of the *Amorphognathus tvaerensis* zone in Sweden, related to the magnetostratigraphy by the conodont zonations (but it has a large 2*σ* confidence interval of 2.1 Myrs; Fig. 7b). The Gullhögen section data produce very different scale to age relationships in the younger and older parts of the optimized composite (Fig. 7b), since it has thinner normal magnetozones over the MO1n and MO1r intervals, and is the only section that constrains the relative thickness of MO1r.1r. The age model uncertainties for the mid–late Ordovician GPTS show larger 95% confidence intervals on chron ages compared with those for the Carboniferous and Permian due to the paucity of age control (Supplementary Tables 5–7).

The late Cambrian and earliest Ordovician have a paucity of radiometric dates^52^, and only two can be directly related to the available magnetostratigraphy (Fig. 7a and Supplementary Table 4). These come with much larger analytical uncertainties than stratigraphic uncertainty. The proposed linear relationship between optimized scale and age is compatible with the base of the Guzhangian Stage at *ca*. 494.4±3.5 Myr (ref. 52; Fig. 7a), an age relationship suggested by the carbon isotope data from the Kulyumbe section in Siberian (Supplementary Fig. 2).

## Discussion

**Figure 8:**
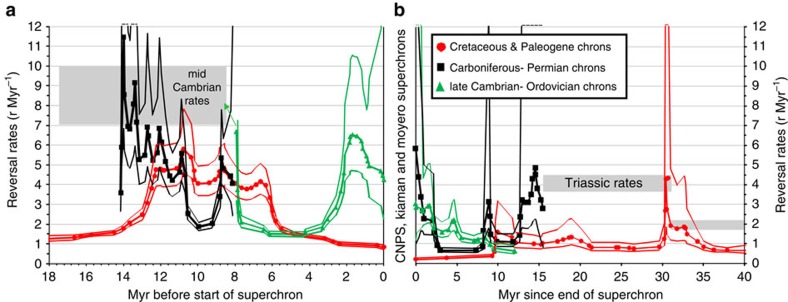
Smoothed reversal rates and 95% confidence intervals. Reversal rates determined using local regression techniques^62^. (**a**) Before the superchrons (0 Myr=superchron start) and (**b**) after the superchron (0 Myr=superchron end). Cretaceous data from ref. 63. Typical mid Cambrian reversal rates^40^ and Triassic rates^56^ shown as grey bars in **a**,**b**. Chron ordinal-age relationships, from which the reversal rates are derived, are shown in Supplementary Fig. 7. The three time intervals coloured accordingly. Confidence intervals use the point-wise method^62^ based on the ordinal-age gradient determination.

Initial post-Kiaman and post-Moyero polarity-reversal rates are ∼2.5–4 r/Myr—rates that are larger than those in the first 30 Myr following the CNPS (Fig. 8b). This shows that, unlike the CNPS, the reversal rates following the Palaeozoic superchrons have an initial fast restart. However, some 4–10 Myr following the Palaeozoic superchrons, reversal rates did decline and were comparable to the lower rates following the CNPS (Fig. 8b). Before all superchrons, reversal rates generally decline from higher values (some 10–15 Myr) before the superchron start, although the Carboniferous data are incomplete (Fig. 8a), and there is some cyclicity in the reversal rates. In the interval 7–15 Myr prior to the initiation of the three superchrons, reversal rates seem to get progressively larger back in time. The same may be the case for the 2 Myr prior to the superchron (Fig. 8a). If the heatflux change across the CMB provides the threshold for entering or leaving the non-reversing state (as suggested by geodynamo models^2^), then the starting and ending of the Palaeozoic superchrons imply either a more rapid change in the heatflux or progressively higher heat flow threshold for older superchrons when entering or leaving the superchron state. This suggests that for geodynamo models to effectively model superchrons through the Phanerozoic, they need to include elements of slow progressive thermal evolution (perhaps via inner core growth or differing thermochemical piles in the lower mantle), as well as the interaction with mantle dynamics.

Some hypotheses of superchron start and end utilize support from the asymmetry in reversal rates either side of the CNPS, since this matches the modelled slow growth (superchron end, lower reversal rates) and faster collapse (superchron start, faster reversal rates) of superplumes^5^. Rate asymmetry can be considered over different time windows. For a *ca.* 2 Myr time window prior and following the CNPS and the Moyero superchrons, there is evidence for higher reversal rates at the superchron start (Fig. 8). The same can be inferred for a *ca.* 15 Myr time window either side of the CNPS and Moyero superchrons. Confirmation of this asymmetry for the Kiaman superchron requires more magnetic polarity data for the 8 Myr before the superchron start (Fig. 8a). However, these data do tentatively suggest that the superplume model may be a viable hypothesis for generating the low-frequency alternation between non-reversing and reversing states in the Palaeozoic.

**Figure 9:**
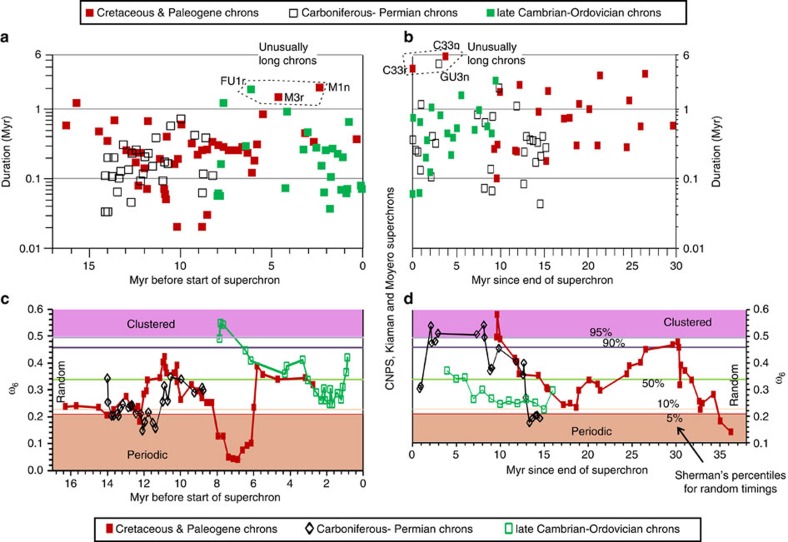
Chron durations and chron patterns. Chron durations (**a**,**b**) and Shermans *ω*_6_ statistic (**c**,**d**), using a six-chron duration sliding window^53^, showing classification of chron intervals into periodic (approximately equal duration), random and clustered (two groups of duration). The *x* axes are scaled in Myr from the older (**a**,**c**) and younger (**b**,**d**) boundaries of the three superchrons. Chron ages, durations and confidence intervals shown in Supplementary Tables 5–7.

The CNPS, Kiaman and Moyero superchrons are 42.3, 51.7 and 19.0 Myr in duration, respectively. The Permian chron GU3n is a long chron (∼4.3 Myr duration) like the Cretaceous C33r (3.7 Myr) and C33n (5.6 Myr), making these three chrons the longest known in the Phanerozoic (apart from the superchrons; Fig. 9b)—all within 9 Myr of the superchron end. These long chrons give rise to large Sherman's *ω*_6_ values^53^, produced by significant groupings (that is, clustered; Fig. 8d) in chron durations. As suggested previously for C33r and C33n (ref. 7), these exceptionally long chrons may be better associated with the superchron geodynamo state, in a transitory behaviour, or ‘memory-related' relict of the previous superchron.

Before the superchrons, there is also a small cluster of long-duration chrons (M1n, M3r, FU1r, all >1.5 Myr in duration) within *ca.* 7 Myr of the superchron start (Fig. 9a). In the Cambrian data, this is shown by the low reversal rates in the 3–7 Myr interval before the start of the Moyero superchron (Fig. 8a). These pre- and post-superchron data together suggest that there is a *ca.* 10 Myr window either side of the superchron in which there is an enhanced likelihood of an unusually long chron occurring, that is, a transitional state either side of the superchron. A speculative suggestion is that the post-superchron transitional state may also be evidenced in ‘memory effects' seen in the structure of the GPTS in statistical modelling^54^,^55^. As suggested for the Cenozoic and Mesozoic^53^, a chron-duration structure identified by Sherman's statistic is seen in the Palaeozoic data, with cyclical swings between periodic and clustered chron durations (Fig. 9c,d). In numerical dynamo simulations, similar changes in Sherman's statistic can be reproduced when control parameters are time-dependent^53^. These cyclical changes through the Phanerozoic are on a timescale of 10 Myr (ref. 53), similar to the duration of the superchron transitional states, both of which may reflect another unknown process that has an impact on the shorter-term polarity-reversal structure of the geodynamo.

## Methods

### Construction of an optimized GPTS

**Figure 10:**
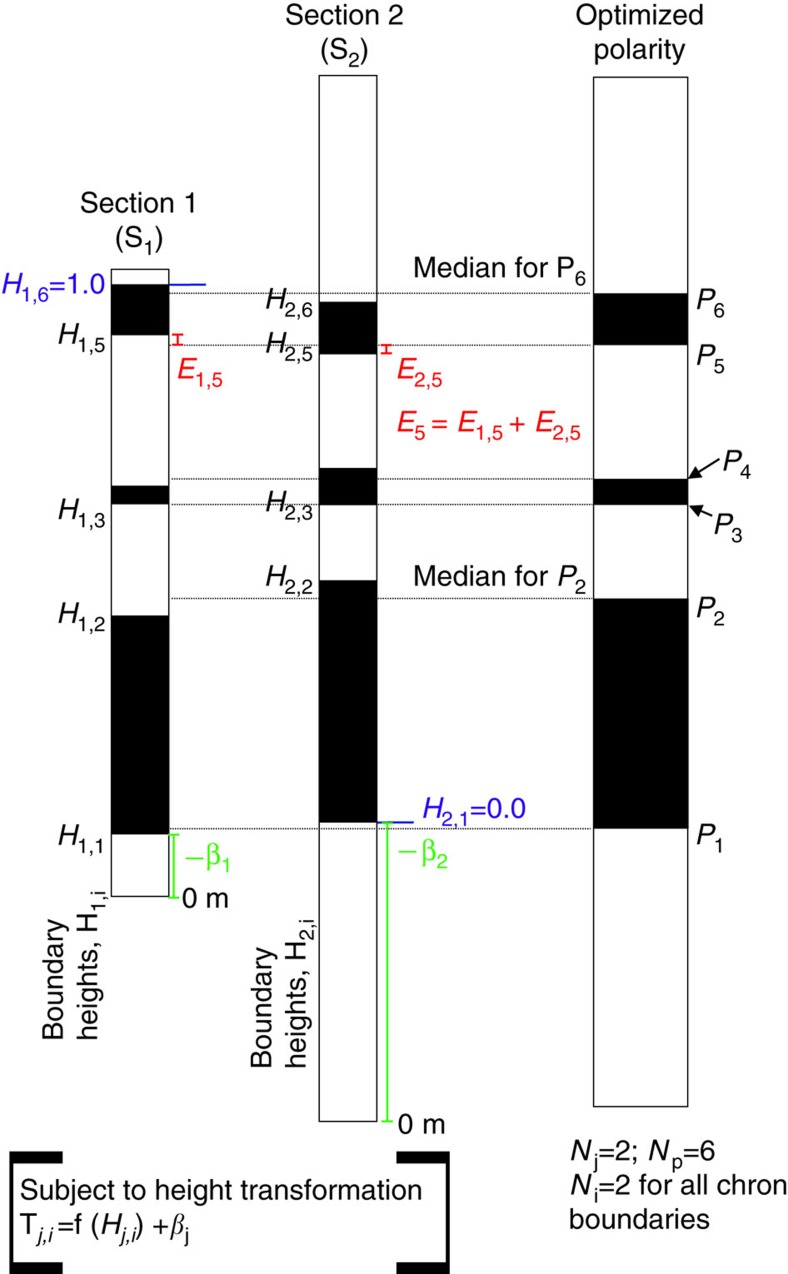
Nomenclature used in the chron optimization method. Data from two hypothetical sections, with magnetozone boundary heights (H_*j,i*_) with respect to the zero level of each section. Shown in red is the individual section *E*_*j*,5_ values (*E*_*j,i*_ is the squared offsets, equation 1) used to measure the residuals from the optimized composite level of the fifth chron boundary (*P*_5_). The medians of the magnetozone boundary levels (horizontal lines) give *P*_*i*_ for each chron. Shown in blue are the positions of magnetozone boundaries that scale the two sets of *H*_*j,i*_ data to a nominal 0–1 scale. The transformation shift offsets for each section (*β*_*j*_) serve to best align all the equivalent magnetozone boundary levels between sections. The actual heights in the two sections are also subject to the rate transformation f(*H*_*j,i*_), producing transformed relative heights of *T*_*j,i*_, which are used to determine the chron positions *P*_*i*_ in the optimization procedure. The example illustrated shows only linear rate transformations.

The optimized composite comprises the relative height of magnetozone boundary, **P**_**i**_, in a succession of *N*_*p*_ magnetozone boundaries (that is, magnetozone boundaries *i*=1 to *N*_*p*_; for example, base of GU1n to base LT1n for the Permian), from the N sections of data (Fig. 10). The height of magnetozone bases is used. From each original section, *S*_*j*_ (*j*=1 to *N* sections), magnetozone boundary *i* will have relative height in that section of **H**_*j,i*_. A measure of the mismatch, **E**_*j,i*_ (Fig. 10), between the position of the magnetozone boundary in the optimized composite (that is, **P**_**i**_) and the equivalent boundary in each section is (**P**_*i*_**−H**_*j,i*_). Summing across all sections containing this magnetozone (*N*_*i*_) gives the total mismatch of:


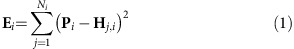


A section-dependent rate transformation f(**H**_*j,i*_) and height shift (*β*_*j*_) was applied to the magnetozone heights in each section, giving new transformed heights **T**_*j,i*_, where **T**_*j,i*_=f(**H**_*j,i*_)+*β*_*j*_ (Fig. 10). This transformation is in affect a sedimentation rate function, and *β*_*j*_ is a shift in the height applied to all magnetozones in that section. In hand-drawn composites^56^ these two factors correspond to a linear stretch (or shrinkage) of the magnetozone scale of each section, and a relative height shift that visually best matches the magnetozone anchor points used in the correlation diagrams. Using these transformations, and summing across all sections containing this magnetozone, gives the residual in the chron mismatch, **E**_*i*_, expressed as:


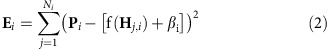


In practice, the number of magnetozone boundaries contributing to **E**_*i*_ for each chron will not be the same throughout the composite. To remove this bias, the value of **E**_*i*_ was normalized by the number of magnetozones (*N*_*i*_) that contribute to **E**_*i*_, which will be ≤*N*; therefore,


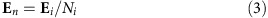


If there are *N*_*p*_ total magnetozone boundaries in the optimized composite, then an expression that represents the sum of the mismatch across all sections and magnetozone boundaries is:


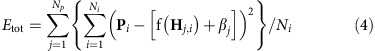


The value of **P**_*i*_ is some average (a median was used here, since there are small numbers of equivalent magnetozone boundaries) of the relative heights of the magnetozone *i* in the *N*_*i*_ sections, subject to the unknown rate transformation and height shift operations (that is, unknowns are f(**H**_*j*_) and ***β***_*j*_). For a perfect set of rate transformation and height shift values *E*_tot_=0. The unknowns for this system can be solved by numerical optimization, which attempts to minimize the value of *E*_tot_. This also determines the minimum **E**_*n*_ values for each magnetozone. The values determined by the optimization are the parameters of the rate transformation f(**H**_*j*_) and the constant *β*_*j*_ for each section (that is, minimum of two unknowns per section, see below).

A second proxy (in addition to **E**_*n*_) for the chron mismatch is the s.d. of **T**_*j,i*_ for each individual magnetozone (that is, *σ*_*T*_; Fig. 5). *σ*_*T*_ was scaled to Myr (from the final age model) to give an uncertainty estimate of the position of the chron.

### Sedimentation rate transformations

Without independent evidence of sedimentation rates for sections, derived perhaps from sedimentological data, or other means of rate determination, a simple and widely used assumption is a constant sedimentation rate in each section^57^. This assumption was used for most sections, since there is often insufficient information for most sections about the sedimentology or accompanying radiometric dates to properly evaluate any sedimentation rate changes. However, different rate functions were used on some sections, showing poorer model fits (Permian nonlinear ones shown in Supplementary Fig. 6). The additional functions used were either simulating increasing sedimentation rate upwards through the section (simulating transgressive sequences) or decreasing sedimentation rates (simulating regressive sequences). The transformation functions used were either constant, transgressive or regressive. For a constant rate, a transformation **T**_*j,i*_=*α*_*j*_ × **H**_*j,i*_+*β*_*j*_ (where *α*_*j*_ is in effect the sedimentation rate constant for each section, where *α*_*j*_>0). For a transgressive rate, simulating smoothly increasing sedimentation rate, **T**_*j,i*_=*β*_*j*_+*α*_*j*_ × F_*j*_(**H**_*j,i*;_ *λ*_*j*_) (where F(*x*; *λ*) is the cumulative exponential distribution function with *λ*≥0). For the regressive rate, simulating smoothly decreasing sedimentation rate, **T**_*j,i*_=*β*_*j*_+*α*_*j*_/F_*j*_(**H**_*j,i*;_ λ_*j*_) (where F(*x*; *λ*) is the exponential distribution probability density function with *λ*≥0 and where *α*_*j*_>0).

Therefore, the unknowns were: which of the rate transformations to use and the values of *α*_*j*_ and *β*_*j*_ (and *λ*_*j*_ if a non-constant rate function is used). Decisions on selecting rate functions depend on evaluating the magnetostratigraphic or geological data (helped by statistics outlined below). The *N* sets of constants *α*_*j*_, *β*_*j*_ (and potentially *λ*_*j*_) are derived numerically by the optimization, giving up to a maximum of three unknown variables for each section.

### Optimization of the rate constants

Minimization of *E*_tot_ was performed using the Solver function in Microsoft Excel (Supplementary Fig. 8). However, to solve this two equality constraints are necessary to fix the vertical extent of the scale for **P**_*i*_, an upper and lower limit (*L, U*). These limits were located at selected magnetozone boundaries in appropriate sections that show the upper and lower limits of the magnetic polarity pattern clearly (Supplementary Tables 8–10). For the Permian data these were the base of GU1n at Monastryki and base of LT1n at Shangsi (Figs 2 and 3). For the late Ordovician these were the base of UO1n.2n and base of LO1r.1n at the Rozhkova section (Fig. 4). For the Carboniferous and late Cambrian–Ordovician data sets, these are shown on Supplementary Figs 1 and 2. The *L* and *U* values in the appropriate spreadsheet cells are keyed into Solver as constraints on the optimization (Supplementary Notes).

The initial **H**_*j,i*_ values were derived from Coreldraw correlation charts (Figs 2, 3, 4 and Supplementary Figs 1 and 2). The **H**_*j,i*_ values were then re-scaled using *L* and *U* to produce new **H**_*j,i*_ values ranging from ∼0 to ∼1 (see Supplementary Data). The optimization yields new transformed height values (**T**_*j,i*_) for each magnetochron boundary in each section. The median of the **T**_*i*_ values (for each magnetochron boundary) was used as an estimate of **P**_*i*_, the magnetochron position in the optimized composite, since there are relatively few data points per magnetochron (Supplementary Tables 8–11).

### Goodness-of-fit statistics of the optimized composite GPTS

The residual between each magnetozone boundary in each section and the optimized composite is **T**_*j,i*_–**P**_*i*_, and the average mismatch in each section per magnetozone is


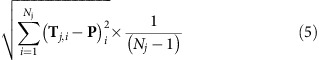


*N*_*j*_=number of magnetozone boundaries in section number *j*; (*N*_*j*_−1) in equation 5, since one more polarity boundary than magnetozones.

However, since the expectation is that the size of the mismatch is related to magnetochron duration^57^, a better measure of relative (that is, between-section) mismatch is to normalize it by the average transformed chron height in that section. Therefore, a measure of the mean mismatch, **D**_*j*_, between the optimized composite and the magnetozone boundary data in each section is:


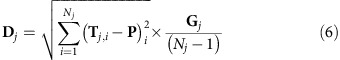


where **G**_*j*_ is the geometric mean of the transformed chron height in the composite, over those chrons which occur in the section. The geometric mean, because chron durations (and so magnetozone-relative heights), vary enormously in relative scale. **D**_*j*_ expresses the proportion of chron mismatch with respect to the mean magnetozone height in the section, and typically ranges from ∼0.1 to ∼0.3 (Supplementary Tables 8–11), with larger values indicating worse matches. For these data sets **D**_*j*_ greater than ∼0.25 indicates a larger than usual misfit. In addition, across all sections


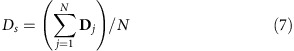


*D*_*s*_ provides a mean measure of the misfit of the entire set of section data. The data sets here have *D*_*s*_ values of 0.07–0.28, indicating some 7–28% uncertainty in chron heights in the optimized composites.

For a perfect set of matching section data **T**_*j*_,_*i*_−**P**_*i*_=0 for all chrons (that is, all chron boundaries in a study section would be perfectly aligned with the optimized composite). This difference is amenable to a paired *t*-test, which evaluates if the mean difference is statistically equivalent to zero, that is,

H_o_:


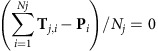


Consequently, values of *t*-test probability, *TP*_*j*_<0.05, would suggest that the **T**_*j,i*_−**P**_*i*_ differences are biased away from zero, rather than being equally distributed about zero. The most likely reason for small *TP*_*j*_ probabilities is that the rate transformation for that section is not well-matched to the optimized chron positions. In this situation, possible alternative rate models were investigated.

Therefore, *D*_*j*_ and *TP*_*j*_ values allow identification of the most problematic section data—that with the largest *D*_*j*_ and/or smallest *TP*_*j*_ pointing to the most anomalous data set. Alternative rate transformations or correlation scenarios investigated were focussed on those section data highlighted by such values.

For each chron boundary, *E*_*n*_ provides a measure of the chron misfit; therefore, larger *E*_*n*_ values flag-up problematic boundaries in the optimized GPTS with a poorer fit. A rather more geologically meaningful parameter than *E*_*n*_ expressing chron misfit is *σ*_*T*_ (Fig. 5), but it is only generated at the age model stage.

### Hiatus and data limits in sections

Additional information is contained in the stratigraphic extent of the last polarity magnetozones at the top and bottom of the sections, since the rate functions applied to the polarity boundaries also apply to these parts of the data. In the Permian Russian data (Fig. 2), the top of the Sukhona section would be expected to be below the base of the overlying normal chron (that is, N_2_R_3_P which is LP2n.3n) in the optimized GPTS. Likewise, the base of the Pizhma section should be above the base of the GU3n chron in the optimized composite. These acted as ‘top and base constraints' in the numerical optimization procedure, even though they may or may not actively control the final chron positions in the optimized GPTS composite. A limited number of these top and base constraints were applied to control the consistency in application of the rate transformations (Supplementary Tables 8–11), and are particularly important for non-constant rate functions.

Magnetozone boundaries defined by a data gap can act as additional ‘mid-section' constraints. The base of magnetozone N_2_P in the Boyevaya Gora section is defined by a data gap; however, the position (in the transformed scale) of the base of N_2_P is likely to be above the base of LP1n in the optimized composite.

Hiatus disrupts application of the rate functions, since sediment (and time) is missing; therefore, applying a rate function across a hiatus is erroneous (but may not result in much additional distortion, if the sediment gap is small compared with the thickness of the section). If the hiatus corresponds with a magnetozone boundary, then the true position of the base of the overlying magnetozone is unknown. In this case, the measured magnetozone base (at the hiatus) acted as a constraint (in effect a base of a subsection within the main section). The base of the equivalent of LP0r in the Nammal Gorge section (Fig. 3) occurs at the hiatus in the Wargal Fm, so that the reverse polarity sample level, immediately above this hiatus, in all likelihood, should be above the base of LP0r in the optimized Permian GPTS. For the late Ordovician GPTS, the well-defined hiatus at the base of the Gullhögen Limestone^58^ allowed the section data to be divided into two subsections (lower and upper Gullhögen), possessing differing rate functions. The constraints applied in the optimized models are shown in Supplementary Tables 8–11.

### Rationale for optimization procedures

In GPTS optimization, the rate transformations started off with linear rate functions, which progressively evolved in some sections to nonlinear rate models, guided by the above statistics. For the Permian data, rate functions were initially assessed using the data in Figs 2 and 3 independently, and then applied to the whole Permian data set (as model P-1 in Supplementary Table 8 and finally P-4 in Supplementary Fig. 6). This assumed more consistency in regional data sets than between regions. Evaluation of *E*_tot_ and *D*_*s*_ identified potentially anomalous sections allowing testing of new rate functions, with the Permian optimization models evolving to the final P-4 model (Supplementary Table 8). The best and simplest optimized GPTS models (lowest degrees of freedom) for the Carboniferous and mid–late Ordovician data sets are using linear rate functions. Nonlinear rate functions were tested for these but gave little or no improvement in *D*_*s*_—an overall rationale that attempted to minimize the *E*_tot_ and *D*_*s*_ values while using the minimum degrees of freedom in the optimization (minimum in unknowns and acting constraints). The best late Cambrian–early Ordovician model uses only nonlinear rate functions. Guidance on using these procedures for other data sets is outlined in the Supplementary Information.

### Radiometric dates, GPTS and reversal rates

The dates were attached to the optimized GPTS using the relative distance from magnetozone boundaries estimated from the individual sections (Supplementary Tables 2–4). The Permian GPTS is constructed from three piecemeal segments, two linear, in the older part, and a spline with generalized validation^59^ in the youngest part of the timescale (Fig. 6b). For the spline, the uncertainty (in Myr) on the U-Pb dates was weighted (1/*σ*^2^_*A*_) using both the uncertainty (*σ*_*R*_ ) in the U-Pb dates (including uncertainty in tracer calibration and *λ*^238^U) and the stratigraphic uncertainty (±*e*_*s*_) in placing the U-Pb date on the magnetostratigraphy, an approach used in other timescales^60^. ±*e*_*s*_ was estimated initially relative to a chron adjacent to the date position. A final ±*e*_*s*_ (in Myr) was determined by placing each U-Pb date on the optimized magnetostratigraphy scaled to Myr. For final spline segment fitting, ±e_*s*_ was converted to s.d. *σ*_*s*_ by *σ*_*s*_=√(*e*_*s*_^2^/12) and the *σ*_*A*_ used for weighting is given by *σ*_*A*_=*σ*_*s*_+*σ*_*R*_ (ref. 60). Linear age models used regression with errors in age (*σ*_*R*_) and stratigraphic uncertainty (±*e*_*s*_) in optimized level units^61^ (Fig. 6). The Carboniferous, mid-Ordovician and late Cambrian–early Ordovician GPTS used only linear age models between radiometric ages and the optimized scales. The uncertainty in the age of magnetochron boundaries was determined by linear regression of each date against its estimated age, the procedure used in the geologic timescales^60^, giving a confidence interval for the chron ages (*C*_95_), using the lower and upper 95% confidence bounds from the regression (Supplementary Tables 5–7). For the late Cambrian–Ordovician GPTS confidence intervals on chron ages could not be determine because of having only two age control points (Fig. 7a).

Polarity-reversal rates (Fig. 8) were determined by evaluating 1/gradient of the chron ordinal versus age relationship (Supplementary Fig. 7 and Supplementary Tables 5–7), using the method of local regression and likelihood as implemented in the LOCFIT routines^62^ in R. Local quadratic polynomials were used for smoothing, with bandwidth selection using generalized cross-validation, and CP_*p*_ statistics^62^. In this, the weighting of the chron ages used were 1/*σ*^2^ derived from the confidence interval C_95._ The late Cambrian-early Ordovician GPTS reversal rates used 1/*σ*_*T*_^2^, for weighting since this age slice has no C_95_ values. Cretaceous chron confidence interval estimates exist for the post-CNPS interval, but not for the pre-CNPS interval^63^. Confidence intervals were estimated from the gradients and the reciprocol provided confidence intervals on the reversal rates.

### Data availability

The author declares that the numerical data supporting the findings of this study are included in the Supplementary Information Data file, in Microsoft Excel format, along with literature sources of the primary data in Supplementary Table 1.

## Additional information

**How to cite this article:** Hounslow, M.W. Geomagnetic reversal rates following Palaeozoic superchrons have a fast restart mechanism. *Nat. Commun.* 7:12507 doi: 10.1038/ncomms12507 (2016).

## Supplementary Material

Supplementary Information Supplementary Figures 1-8, Supplementary Tables 1-11, Supplementary Note 1 and Supplementary References

Supplementary Data 1 Excel file with source data, and notes on its use
