# Supplementary material for: Geomagnetic reversal rates following Palaeozoic superchrons have a fast restart mechanism
Source: Nat Commun. 2016 Aug 30;7:12507. doi: 10.1038/ncomms12507 (PMC5013562; doi:10.1038/ncomms12507)
Supplement: Supplementary Information — Supplementary Figures 1-8, Supplementary Tables 1-11, Supplementary Note 1 and Supplementary References [file ncomms12507-s1.pdf]

# Supplementary Information

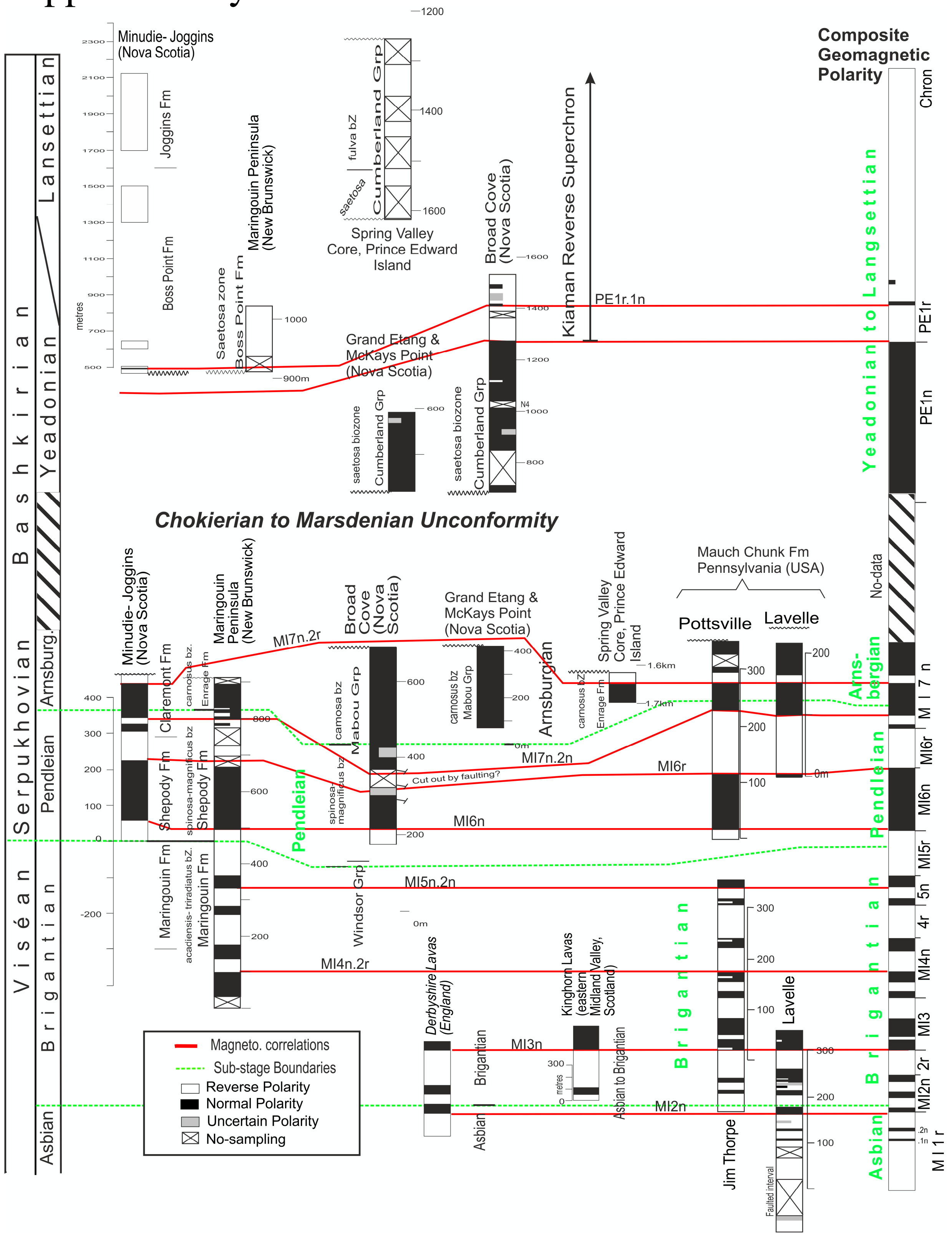


Supplementary Figure 1. Summary magnetostratigraphic data through the Mississippian- Pennsylvanian boundary. Compiled from American sections[1] and European sources[2] (additional details in Supplementary Table 1). Section thicknesses (m) indicated in all but the Derbyshire Lavas. Chron numbers are prefixed by MI (Mississippian), and PE (Pennsylvanian).

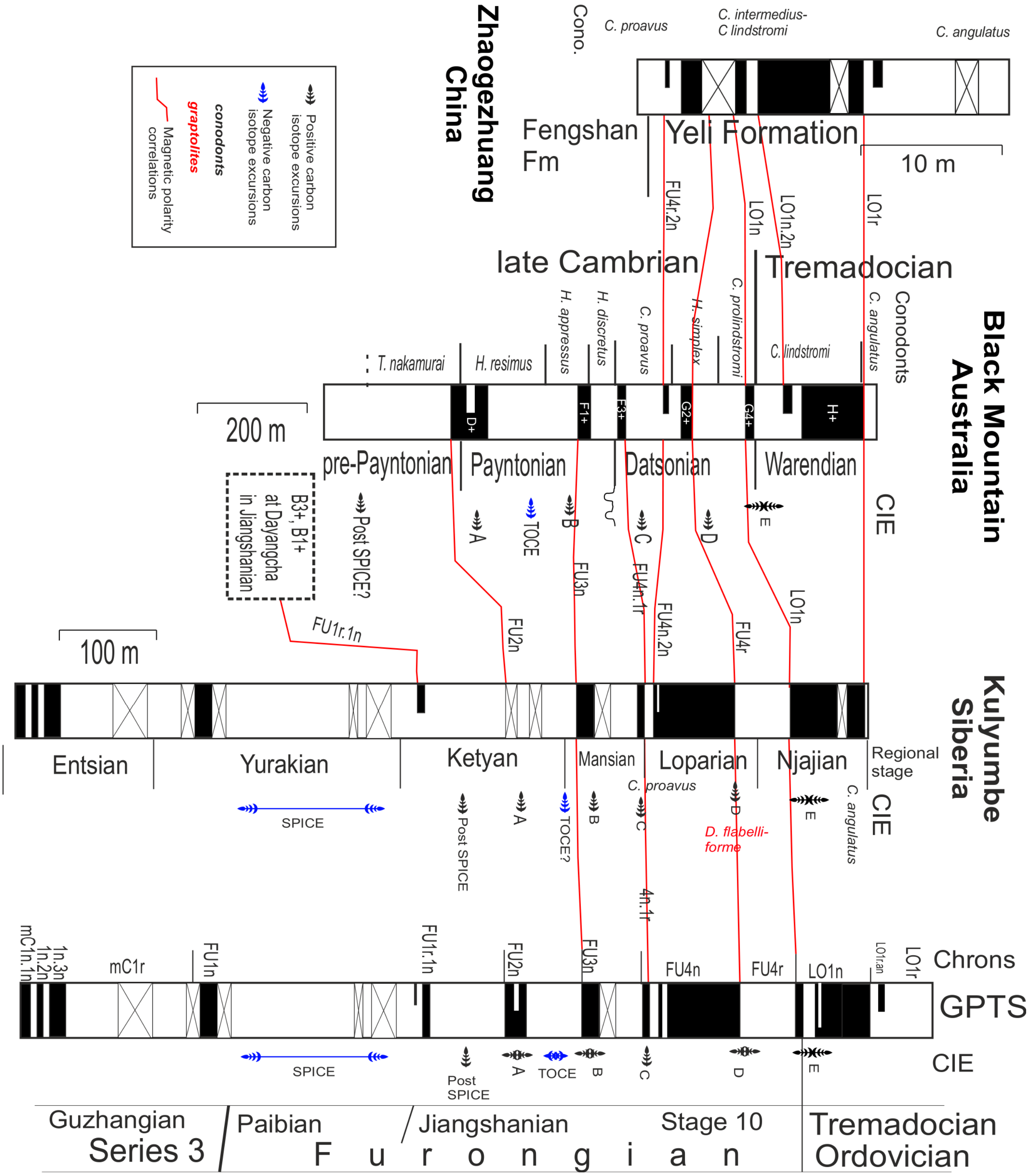


Supplementary Figure 2. Summary magnetostratigraphic data through the late Cambrian- Ordovician boundary. Magnetostratigraphy compiled from sources indicated in Supplementary Table 1. Carbon isotope excursion (CIE) data[3], are labelled with the isotope names (SPICE, TOCE from Ref. 4), with stages related to isotope stratigraphy based on Refs. 3-8. The carbon isotope positive excursions (A to E) above the SPICE negative excursion have been labelled arbitrarily, using relationships shown in Refs. 3,8. The polarity stratigraphy is not shown for the Dayangcha section[9] since the data is fragmentary, and broken by hiatus, although the section data do validate chron FU1r.1n, with an additional tentative magnetozone (FU1r.an) around this level. Chron numbers are prefixed by mC (informal mid Cambrian), FU (Furongian) and LO (Lower Ordovician).

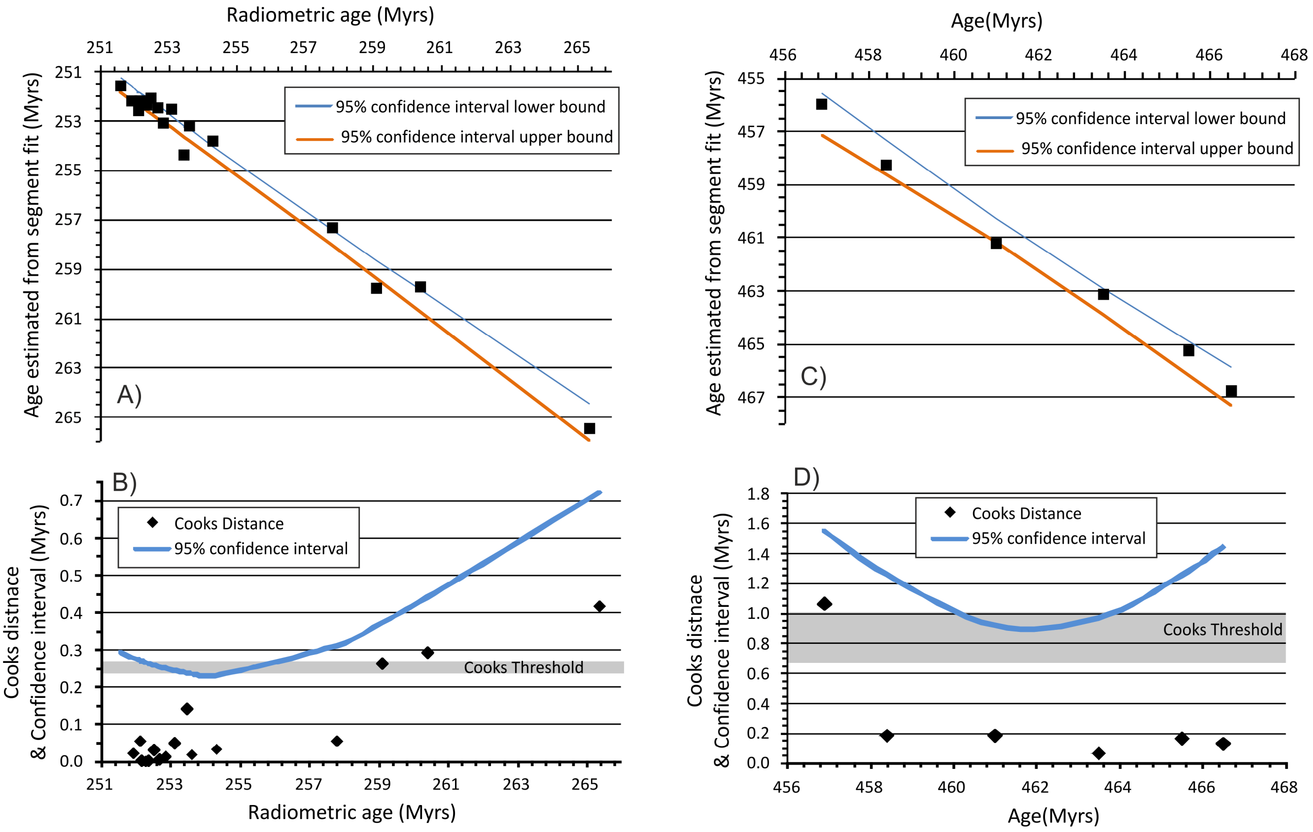


Supplementary Figure 3. Age model statistics for the Permian (A, B) and the mid-late Ordovician (C, D) timescales. A and C show the 95% confidence intervals on the chron ages derived from a linear regression of age tie points, versus the predicted age of the level at which the age points occurs in the magnetostratigraphy[10]. B), D) show the resulting 95% confidence interval (in blue) on chron ages, and the Cooks distance (measure of the relative contribution to the regression, if this point is removed[11]) for the dates, derived in A and C.

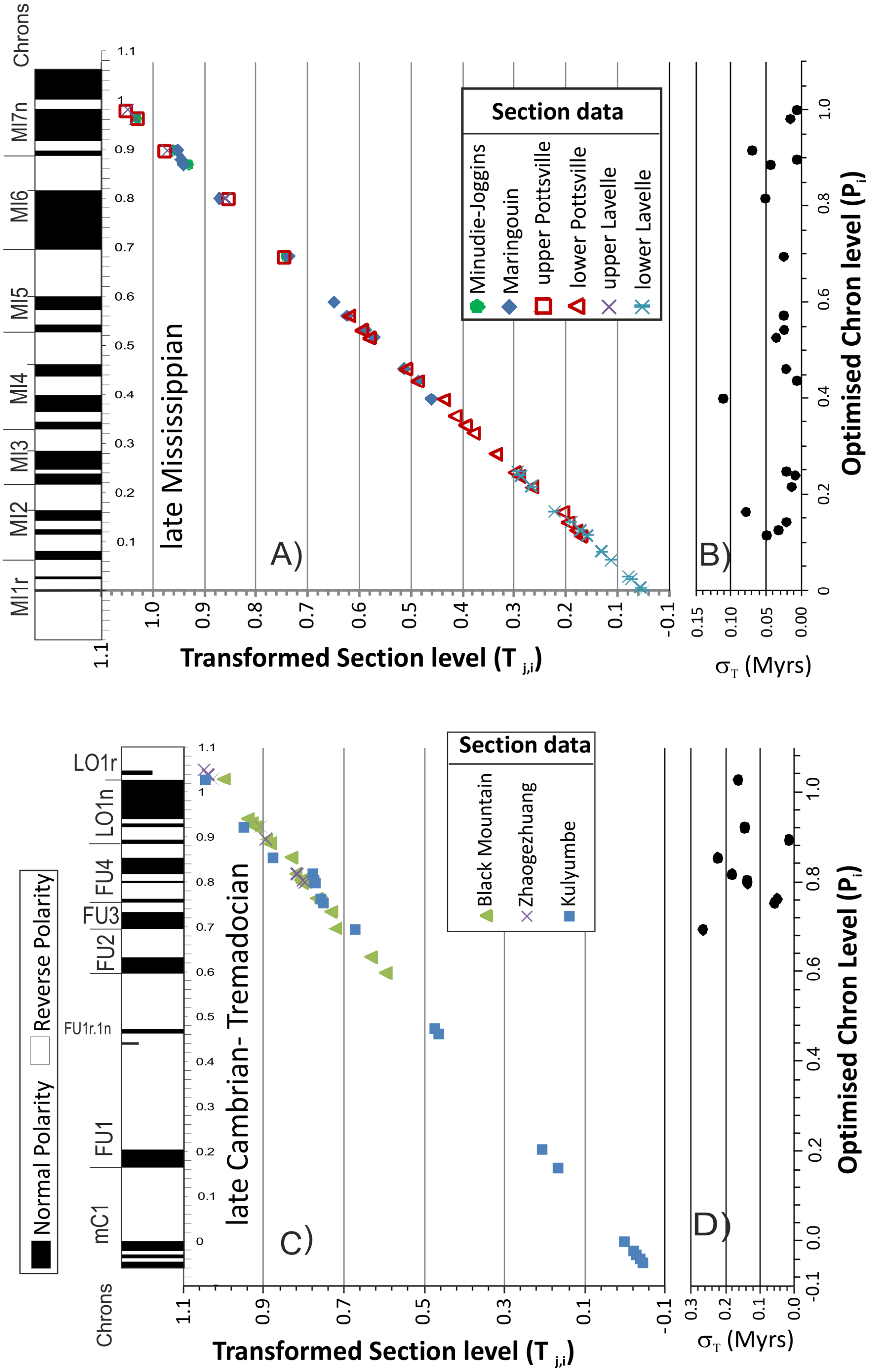


Supplementary Figure 4. Optimised reversal stratigraphy (A,C) for the Mid Carboniferous (late Mississippian) and late Cambrian- Lower Ordovician datasets. A) and C) the base of the transformed corresponding magnetozone from each section is shown on the x-axis, along with the median chron level (**P**$_i$) on the y-axis. B) and D) show $\sigma_T$ - the standard deviation of the data for chron $T_i$ values displayed on the x-axis (in transformed units). The standard deviation of $\mathbf{T}_i$ is scaled to Myrs, using the chron durations from Supplementary Tables 6 & 7.

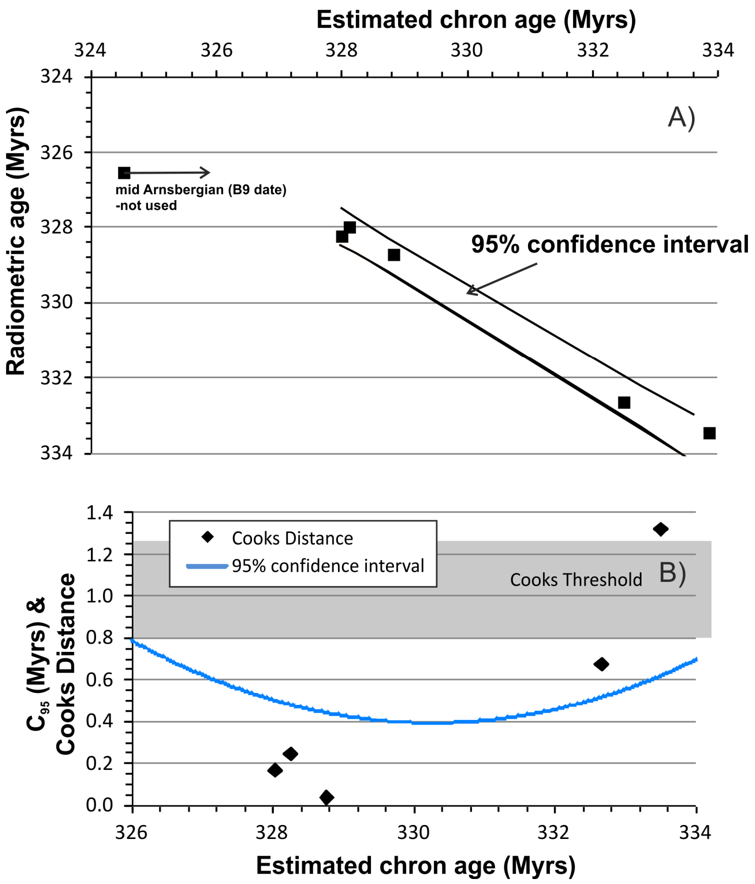


Supplementary Figure 5. Age model statistics for the Carboniferous timescale. A) The 95% confidence intervals on the chron ages derived from a linear regression of age tie points, versus the predicted age of the level at which the age points occurs in the magnetostratigraphy. The unused B9 radiometric date (Supplementary Table 3) illustrates the position of the mid Arnsbergian.  B) The resulting 95% confidence interval (in blue) on chron ages, and the Cooks distance (measure of the relative contribution to the regression, if this point is removed) for the dates, derived in A).

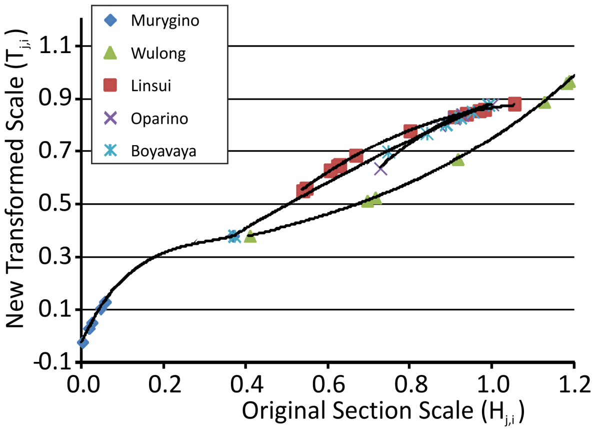


Supplementary Figure 6. The Permian model P-4 showing the non-constant rate transformations (Supplementary Table 8). The transformation of the magnetozone boundaries expressed in the $\mathbf{H}_{j,i}$ scale and the transformed scale ($\mathbf{T}_{j,i}$) derived by optimisation. Showing the transgressive (Murygino, Linsui, Oparino, Boyavaya Gora) and regressive transformations (Wulong), displayed as convex-up and convex downwards curves respectively. Constant rate transformations between $\mathbf{H}_{j,i}$ and $\mathbf{T}_{j,i}$ of all other Permian sections not shown.

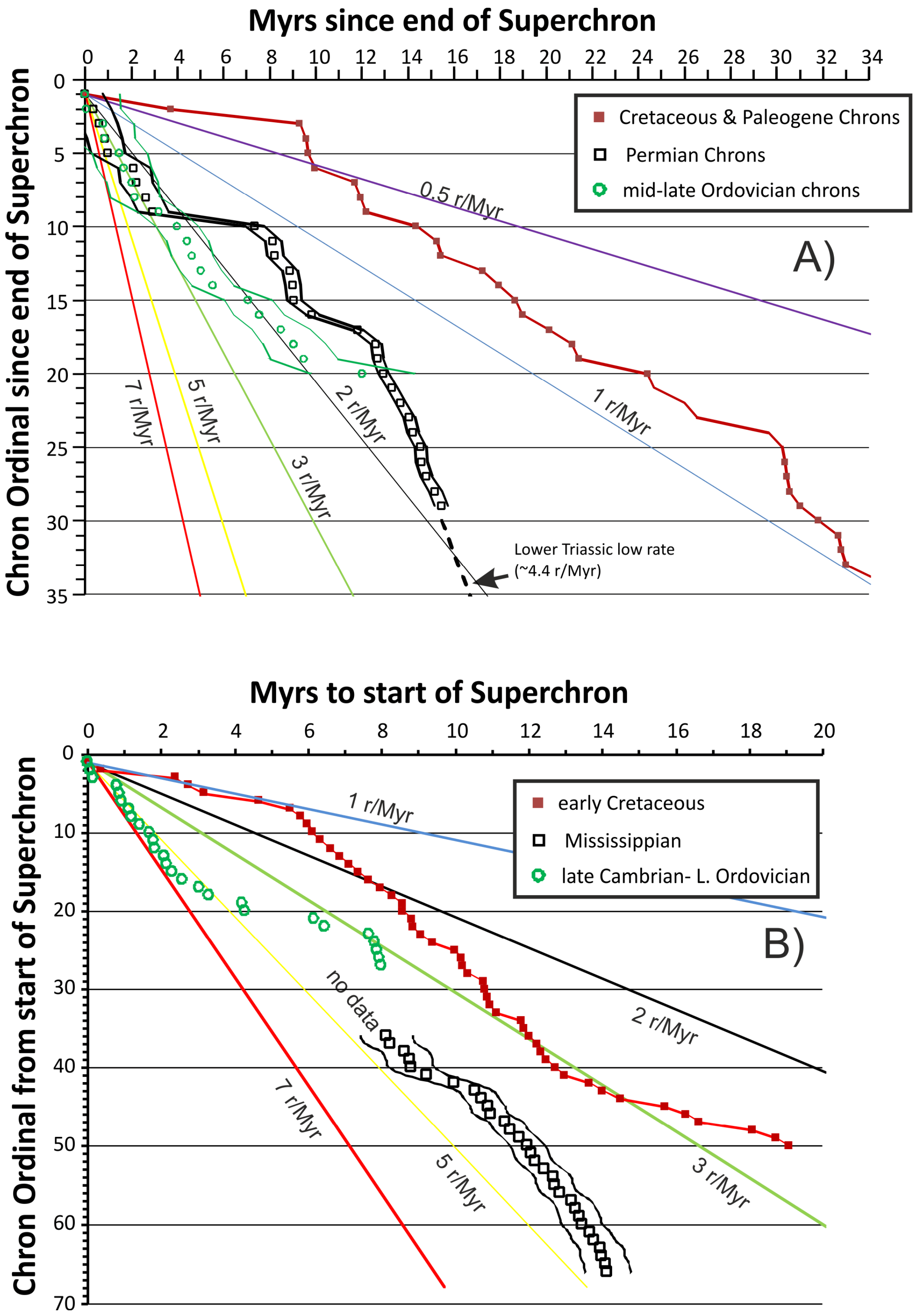


Supplementary Figure 7. Chron ordinal (i.e. chron number) versus age of base of chron for: A) the post superchron intervals, B) the pre-superchron intervals. In A) ordinal 1 is the first chron following the superchrons, in B) ordinal 1 is the first chron prior to the superchron. Cretaceous and Paleogene data from Ref. 12. 95% confidence interval shown on data derived here for the Permian and mid-late Ordovician data (black and green lines in A), and on the Mississippian data (black in B). No confidence interval is derived from the late Cambrian- Lower Ordovician dataset. Reversal rates shown in colour. Projected possible minimum rates shown for the Lower Triassic[13].

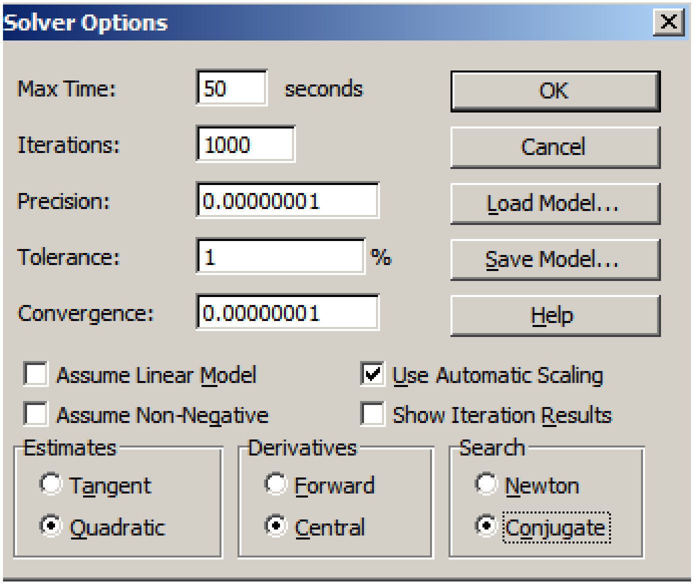


Supplementary Figure 8. The options used in Excel Solver v.2007**.** Estimates, derivatives and search method settings can be modified to come to a final stable solution.

Supplementary Table 1. Summary of the data sources used in the composite magnetostratigraphies and other sources of supporting stratigraphic details.

| **Sections, country** | **Magnetostratigraphy Source References** | **Additional reference sources of biostratigraphy and stratigraphy** |
|---|---|---|
| **Permian, Russian data** | | |
| Khei-Yaga | [14] | [15] |
| Murygino, Pizhma Oparino | [16] | [17,19,20] |
| Tetyushi, Cherumuska, Pityatino | [16] | [17,19,20] |
| Boyevaya Gora, Tuyembetka, Sambullak | [18] | [21] |
| Sukhona River | [22] | [17,19,20] |
| Monastyrski, Westfhal composite | [16,23,24] | [17,19,20] |
| **Permian, Marine and Chinese data** | | |
| W.Texas/New Mexico, USA | [25] | [26-28] |
| Meishan Composite, China | [30-32] | [33-38] |
| Linshui, China | [39] | [40] |
| Ebian County, China | [41-44] | [45,46] |
| Wulong, China | [39, 47] | [48] |
| Shangsi Composite, China | [49-51] | [13,33-35,48,52,53] |
| Taiyuan, China | [54] | [55,56] |
| Nammal Gorge, Pakistan | [57] | [58-62] |
| Abadeh, Iran | [63] | [64,65] |
| **Mid Carboniferous Data:** | | |
| Pottsville, Lavelle, USA | [66] | |
| Minudie-Joggins, Nova Scotia, Canada | [1,67] | [68] |
| Broad Cove, Grand Etang, Nova Scotia, Canada | [1] | [68] |
| Maringouin Peninsula, New Bunswick, Canada | [1,69] | [68] |
| Spring Valley, Prince Edward Island, Canada | [1] | [68] |
| Derbyshire and Kinghorn, UK | [2] | |
| **Late Cambrian-Ordovician Data:** | | |
| Mójcza, Poland | [70] | [71,72] |
| Gullhögen, Sweden | [73] | [74-78] |
| Polovinka, Russia | [79] | [80-81] |
| Rozhkova, Russia | [82] | [80-81] |
| Moyero, Kudrino & Alexeevka, Russia | [83] | [89-81] |
| Kulyumbe, Russia | [3,84] | [4,6-8] |
| Black Mountain, Australia | [85] | [5] |
| Zhaogezhuang, China | [86] | |

Supplementary Table 2. Permian radiometric ages used. **Column 1:** Analysis code and date (in Myr ago). **Column 2:** $\pm 2\sigma_R$ = two-sigma error on age, including uncertainty on tracer calibration and $\lambda^{238}$U. **Column 3**: $\pm e_s$= estimated stratigraphic error (in chron relative duration) in placing the radiometric age onto the magnetostratigraphy[10]. **Column 4:** section name, location and sample location. **Column 5:** Stratigraphic age or location and correlated chron position of radiometric date, measured from base of chron. **Column 6:** Cooks distance, a measure of the how much the residuals from the other dates would change if this date were excluded from the age model- bigger values indicate a greater discordance. Here, a value above ~0.25 indicates the point may be unduly influencing the regression. **Column 7:** source reference for the radiometric information. At Shangsi the first occurrence of *H. parvus* is 4.5 m above the top of the Dalong Fm[51], but only 3.8 m according to Supplementary Fig. 4 in ref. 36, and so the 100 m level appears misplaced downwards with respect to the log in ref. 36, clearly seen by fact PTB is within the apparent Dalong Fm. At the Shangsi section ref. 36 takes the PTB to be at the base of the *H. eurypyge* conodont zone. Hence, the Permian-Triassic boundary (PTB) at Shangsi used in ref. 36 should be 0.37m above the base of the Feixianguan Fm. The base of LT1n at Shangsi is at the base of the clastic interval (±0.5m) in the base of the Feixianguan Fm[51], so is some 0.37m below the PTB (first *H. eurypyge* ) used by ref. 36. [..]$^S$= alternate date from same ash bed in ref. 36; [..]$^m$= alternate date from same ash bed in ref. 33.

| **1.Code, age (Myr ago)** | **2. $\pm 2\sigma_R$ (Myr)** | **3. $\pm e_s$** | **4. Location** | **5.Biostratigraphy, stratigraphy {magnetochron relative position} [other, Myr]** | **6. C.d** | **7. Ref.** |
|---|---|---|---|---|---|---|
| B.Bed 33 251.58 | 0.29 | 50% of LT1n.1n | Meishan, bed 33, 1.75 m above PTB | Lower part of *I. isarcica* Zone {top of LT1n.1r} | 0.00 | [87] |
| B-bed-25,28 251.91 | 0.28 | 30% of LT1n | Meishan, beds 25 & 28 combined, at PTB | Span extinction interval, through *H. changxingensis* & *C. zhejiangensis* zones {10% into LT1n.1n}. [ 252.19]$^s$ [252.44]$^m$ | 0.02 | [38,87] |
| B.Bed-22, 252.10 | 0.28 | 20% of LP2r-LT1n | Meishan, Base bed 22, 4.5 m below PTB | Top L10 zone, {40% through LP3r-LT1n interval}. [252.50]$^s$ | 0.06 | [87] |
| S.bed-15, 252.85 | 0.3 | 30% of LP2n.2n - 2n.3n | Meishan, mid bed 15, 17.4 m below PTB | Mid L10 zone {80% into LP2n.2n - .2n.3n} | 0.01 | [36] |
| S.bed-6&7 253.47 | 0.3 | 20% of LP2n.1n | Meishan beds 6, 7 combined, 36.4-35.9 m below PTB, 4.9 -4.4m above base Changhsingian | Mid L8 zone, {70% into LP2n.1n} | 0.14 | [36] |
| SH32 252.5 | 0.5 | 5% of LT1n.1n | Bed 29, Shangsi, in L12 zone?, 1.1 m below FAD *H. parvus*, 3m above base *H. eurypyge* Zone. | Top *H. eurypyge* zone, {42% into LT1n.1n} | 0.03 | [33] |
| SH10+SH09, 252.35 | 0.6 | 10% of LP3r | Shangsi, beds 25 and 27 average, base of LT1n, 0.4 m below base *H. eurypyge* Zone. | Latest Changshingian, {base LT1n.1n} | 0.00 | [33,34] |

| | | | | | | |
|---|---|---|---|---|---|---|
| SH03-12, 252.16 | 0.3 | 5% of LT1n.1n | Shangsi, 0.5 m above base *H. eurypyge* Zone, | base *H. eurypyge* Zone {9% into LT1n.1n}. | 0.00 | [36] |
| SH03-07, 252.28 | 0.3 | 10% of LP3r | Shangsi, 0.2 m below base *H. eurypyge* Zone | Mid L12 zone {base of LT1n.1n}. | 0.00 | [36] |
| SH03-08, 252.37 | 0.3 | 10% of LP3r | Shangsi, 0.3 m below base *H. eurypyge* Zone | Top L11 zone {60% into LP3r}. | 0.00 | [36] |
| SH03-09, 252.68 | 0.3 | 20% of LP3n | Shangsi, 1.0 m below base *H. eurypyge* Zone | mid L11 zone {50% into LP3n}. | 0.01 | [36] |
| SH03-17, 253.10 | 0.3 | 15% of LP2r | Shangsi, 2.9 m below below base *H. eurypyge* Zone, bed 23 | Top L10 zone {90% into LP2r}. [253.24][m]. | 0.05 | [36] |
| SH03-22, 253.60 | 0.3 | 20% of LP2 | Shangsi, 12.8 m below base *H. eurypyge* Zone, top of bed 18 | Mid L9 zone, Wuchiapingian {70% into LP2 interval}. | 0.02 | [36] |
| SH03-18, 254.31 | 0.3 | 20% of LP2 | Shangsi, 17.1 m, below base *H. eurypyge* Zone; 0.9 m below the base of L7), mid bed 18 | Top L8 zone, Wuchiapingian {42% into LP2 interval}. [253.69][m]. | 0.03 | [36] |
| SH03-5, 257.79 | 0.3 | 10% of LP1n | Shangsi, 27.5m below base *H. eurypyge* Zone, top bed 15. | Top L5 zone, Wuchiapingian, {95% into LP1n} [257.30][m]. | 0.06 | [36] |
| SH03, 260.74 | 0.9 | 25% of LP0r | 36.3 m above base Lopingian, Shangsi, bed 8 | Base Lopingian {base of LP0r} | 0.29 | [33,44,88] |
| JW1, 259.1 | 0.5+ | 10% of GU3n | Emeishan basalts, Zhaotong | ~100 m below top of unit III {95% into GU3n} | 0.27 | [43] |
| NH, 265.35 | 0.5 | 40% of GU2n | Below base of Capitanian, Nipple Hill, Guadalupian Mts | Just below base Capitanian {30% into GU2} | 0.42 | [26,88] |

Supplementary Table 3. Carboniferous dates used. **Column 1:** Analysis code and date (in Myr ago). **Column 2:** ±2$\sigma_R$ = two-sigma error on age. **Column 3**: ±$e_s$= estimated stratigraphic error in placing the date onto the magnetostratigraphy in units of chron widths. **Column 4:** section name, location. **Column 5:** Stratigraphic age or location, {..}= correlated chron position of date. **Column 6:** Cooks distance, a measure of the how much the residuals from the other dates would change if this date were excluded from the age model; bigger values indicate a greater discordance. Here a value above ~1.3 indicates the point may be unduly influencing the regression. **Column 7:** source reference for the radiometric and age information. Date from B9 not used for the age model (but shown in Supplementary Figure 5a), since it is just above the available magnetostratigraphy. The start of the Kiaman superchron is taken from the time-scale in Ref. 95, corresponding to the upper Yeadonian.

| **1.Code, age (Myr ago)** | **2. ±2$\sigma_R$ (Myr)** | **3. ±$e_s$** | **4. Location [estimated position]** | **5.Biostratigraphy, stratigraphy {magnetochron relative position}** | **6. C.D** | **7. Ref.** |
|---|---|---|---|---|---|---|
| B9 324.54 | 0.46 | 100% of MI7n.3n | Oakenclough Brook, Pennines basin, England | Namurian E2b2 ammonoid subzone, Mid Arnsbergian {above MI7n.3n?} | - | [89] |
| Karel Coal 328.01 | 0.39 | 50% of MI6n | Staric 2 core, Upper Silesia Basin, Czech Rep. | mid to upper Pendleian, Coal 106, Hrusov Mbr of Ostrava Fm {70% into MI6n} | 0.25 | [90] |
| C11 coal 328.14 | 0.4 | 30% of MI6n | Yuzhno-Donbasskaya, Ugledar, Donets Basin, Ukraine | Middle $C_{1Vg2}$ zone- Foraminifera, *Betpakodiscus cornuspiroides*, correlated with Pendleian *Eumorphoceras* 1 ammonoid zone , {base MI6r} | 0.17 | [91,92] |
| Ludmilla coal 328.84 | 0.41 | 30% of MI6n | Staric 2 core, Upper Silesia Basin, Czech Rep. | early Pendleian, Coal 043, Petrkovice Mbr of Ostrava Fm {base MI6n} | 0.04 | [90] |
| W13 332.5 | 0.40 | 50% of MI3 | Watrisse Quarry, Anhée Sud, Belgium | Lower part of Anhee Fm, in foram. zone MFZ14 (*Howchinia bradyana* interval zone), upper Asbian. {mid MI6r} | 0.68 | [92,93] |
| 02VD-0 333.87 | 0.39 | 60% of MI1r.1r | Base Bed 21-8, Verkhnyaya Kardailovka, Urals, Russia | mid *L. mononodosa* Zone, and near the base of the *Endothyra assymetrica* foraminiferal Zone=Belgium foram. zone MFZ14, Guisiken Fm, upper Asbian {base MI1r.1r} | 1.32 | [93,94] |

Supplementary Table 4. Late Cambrian- Ordovician dates used. **Column 1:** Analysis code and date (in Myr ago). C&S= ref. 97 CONOP date from their Table 20.1. **Column 2:** ±2$\sigma_R$ = two-sigma error on age. **Column 3**: ±$e_s$= estimated stratigraphic error in placing the date onto the magnetostratigraphy in units of chron widths. **Column 4:** section name, location [..]=position used for zonal suite boundary, or conodont biozone. **Column 5:** Stratigraphic age or location, {..}= correlated chron position of date. **Column 6:** Cooks distance, a measure of the how much the residuals from the other dates would change if this date were excluded from the age model; bigger values indicate a greater discordance. Here a value above ~0.8 indicates the point may be unduly influencing the regression. **Column 7:** source reference for the radiometric and age information.

| **1.Code, age (Myr ago)** | **2. ±2$\sigma_R$ (Myr)** | **3. ±$e_s$** | **4. Location [estimated position]** | **5.Biostratigraphy, stratigraphy {magnetochron relative position}** | **6. C.D** | **7. Ref.** |
|---|---|---|---|---|---|---|
| G2012 456.9 | 2.1 | 50% of UO1n.2n | Mossen quarry, Kinnekulle Sweden | Upper *A. tvaerensis* Zone {mid UO1n.1n} | 1.1 | [96], pg. 1072-1073 |
| C&S 458.4 | 0.9 | 50% of UO1n.1n | [Base of *R. inequalis* at Mójzca, Poland]. | From C&S, 2σ uncertainty from GI1 zonal suite, {base UO1n.1n} | 0.19 | [97] |
| C&S 461 | 0.8 | 50% of MO2n.1n | [Base of *E. lindstroemi* subzone at Mójzca]. | From C&S, 2σ uncertainty from Da4 zonal suite, {base MO5r} | 0.19 | [97] |
| C&S 463.5 | 0.8 | 100% of MO1r.1n | [Top of Holen Limestone at Gullhögen, Sweden] | mid *E. suecicus* Zone. From C&S Fig. 20.1. 2σ uncertainty from DA3 zonal suite {base MO1r.1n} | 0.07 | [74,97] |
| C&S 465.5 | 0.9 | 50% of MO1n.1n- .2n | [Base of Holen Limestone at Gullhögen, Sweden] | Mid *Enodus (Eoplacgonathus) variabilis* Zone. From C&S, 2σ uncertainty from DA2 zonal suite, {base MO1n.1n} | 0.17 | [76,97] |
| C&S 466.5 | 1.0 | 100% of LO1n.1n- .2n | [Mid Volginian at lower Rozhkova, Siberian] | Near top *B. norrilandicus* Zone . From C&S, 2σ uncertainty from DA1 zonal suite, {base LO1r.1n} | 0.13 | [76,97,98] |
| O1 486.8 | 2.57 | 50% of LO1n.3n | Bryn-llyn-fawr, Wales [basal *C. lindstromi* zone at Black Mountain] | 1 m below FAD *Rhabdinipora* sp. at Bryn-llyn-fawr, correlated to ranges at Tremadocian GSSP {mid LO1n.1r}. | - | [5,88,99] |
| C11 488.71 | 2.78 | 100% of FU2n | Ogof Ddu, Wales [mid way between CIE peaks A and TOCE at Black Mountain] | Lower half of *Peltura scarabaeoides* Zone, correlated to just below *E. notchpeakensis*, in proposed GSSP for ‘Lawsonian’ Stage {17% into FU2r}. | - | [88,99] |

Supplementary Table 5. Permian and earliest Triassic chron base ages, durations and optimisation statistics. $E_n$ from equation 3 in main text. $C_{95}$ is the 95% confidence interval on the age of the chron (from Supplementary Figure 3). $\sigma_T$ is the standard deviation of the section mismatch (i.e. $\mathbf{T}_{j,i}$) for the chron scaled to the duration of that chron, and is a measure of uncertainty in defining the chron position in the optimised GPTS. $N_i$ is the total number of magnetozones contributing to that chron. Reversal rates determined using a LOCFIT smoothing parameter (alpha) of 0.31, corresponding to a 9 chron bandwidth. Base Lopingian estimated at 259.6 Myr and base Triassic estimated at 252.24 Myr.

| Chron | Age (Myr) | $E_n$ (optimised height units) | Chron duration (Myr) | $C_{95}$ (Myr) | $N_i$ | $\sigma_T$ kyrs | Rev. rates (r per Myr) |
|---|---|---|---|---|---|---|---|
| LT1n.2n | 251.580 | - | | 0.29 | 1 | - | 2.78 |
| LT1n.1r | 251.855 | - | 0.094 | 0.28 | 1 | - | 3.24 |
| LT1n | 252.256 | 0.000013 | 0.320 | 0.27 | 2 | 27 | 3.81 |
| LP3r | 252.466 | 0.000080 | 0.258 | 0.26 | 4 | 61 | 4.52 |
| LP3n | 252.509 | 0.000087 | 0.060 | 0.26 | 4 | 55 | 4.84 |
| LP2r | 252.833 | 0.000185 | 0.357 | 0.25 | 5 | 80 | 4.29 |
| LP2n.3n | 253.003 | 0.000061 | 0.141 | 0.25 | 6 | 73 | 3.50 |
| LP2n.2r | 253.355 | 0.000065 | 0.307 | 0.24 | 7 | 103 | 3.10 |
| LP2n.2n | 253.715 | 0.000159 | 0.328 | 0.23 | 8 | 210 | 3.11 |
| LP2n.1r | 254.112 | 0.000302 | 0.367 | 0.23 | 8 | 358 | 3.23 |
| LP2n.an | 254.349 | - | 0.222 | 0.23 | 1 | - | 2.37 |
| LP2n.ar | 254.433 | - | 0.078 | 0.23 | 1 | - | 1.45 |
| LP2n.1n | 255.206 | 0.000153 | 1.036 | 0.24 | 4 | 383 | 1.10 |
| LP1r | 257.195 | 0.000183 | 1.889 | 0.29 | 4 | 508 | 1.11 |
| LP1n.2n | 257.976 | 0.000187 | 1.290 | 0.32 | 3 | 330 | 1.46 |
| LP1n.1r | 258.042 | 0.000191 | 0.065 | 0.32 | 3 | 104 | 2.38 |
| LP1n.1n | 258.179 | 0.000294 | 0.137 | 0.33 | 9 | 111 | 3.14 |
| LP0r.2r | 258.821 | 0.000322 | 0.642 | 0.36 | 5 | 123 | 1.66 |
| LP0r.1n | 258.893 | 0.000281 | 0.072 | 0.36 | 5 | 115 | 0.86 |
| LP0r | 259.704 | 0.000085 | 0.811 | 0.40 | 8 | 60 | 0.66 |
| GU3n | 264.103 | 0.000170 | 4.399 | 0.66 | 5 | 269 | 0.67 |
| GU2r.2r | 264.421 | 0.000017 | 0.318 | 0.67 | 3 | 100 | 0.91 |
| GU2r.1n | 264.864 | 0.000018 | 0.403 | 0.69 | 3 | 102 | 1.56 |
| GU2r | 264.928 | 0.000017 | 0.104 | 0.70 | 5 | 91 | 2.18 |
| GU2n | 266.073 | 0.000093 | 1.146 | 0.76 | 5 | 214 | 2.28 |
| GU1r.2r | 266.205 | - | 0.131 | 0.76 | 1 | - | 2.67 |
| GU1r.1n | 266.446 | - | 0.242 | 0.77 | 1 | - | 3.37 |
| GU1r | 266.699 | 0.000057 | 0.253 | 0.78 | 5 | 167 | 4.39 |
| GU1n | 267.059 | 0.000083 | 0.360 | 0.80 | 5 | 202 | 5.85 |

Supplementary Table 6. Carboniferous chron base ages, durations and optimisation statistics. Columns as in Supplementary Table 5. Reversal rates determined using a LOCFIT smoothing parameter (alpha) of 0.26, corresponding to an 8 chron bandwidth. Base Brigantian estimated at 332.45 Myr, base Pendleian (and Serpukovian) at 328.93 Myr and base Arnsbergian at 327.35 Myr.

| Chron | Age (Myr) | $E_n$ (optimised height units) | Chron duration (Myr) | $C_{95}$ (Myr) | $N_i$ | $\sigma_T$ kyrs | Rev. rates (r per Myr) |
|---|---|---|---|---|---|---|---|
| MI7n.3n | 326.92 | 0.000000 | 0.371 | 0.71 | 2 | 5 | 4.06 |
| MI7n.2r | 327.03 | 0.000003 | 0.112 | 0.69 | 3 | 14 | 4.43 |
| MI7n.2n | 327.43 | 0.000098 | 0.394 | 0.64 | 4 | 69 | 4.82 |
| MI7n.1r | 327.55 | 0.000000 | 0.118 | 0.63 | 2 | 5 | 5.33 |
| MI7n.1n | 327.61 | 0.000025 | 0.063 | 0.62 | 2 | 43 | 3.37 |
| MI6r | 328.03 | 0.000051 | 0.423 | 0.57 | 4 | 49 | 2.02 |
| MI6n | 328.76 | 0.000010 | 0.729 | 0.51 | 3 | 24 | 1.85 |
| MI5r | 329.33 | - | 0.573 | 0.48 | 1 | - | 2.53 |
| MI5n.2n | 329.50 | 0.000008 | 0.167 | 0.48 | 2 | 23 | 4.50 |
| MI5n.1r | 329.68 | 0.000007 | 0.177 | 0.47 | 2 | 22 | 5.58 |
| MI5n.1n | 329.77 | 0.000016 | 0.095 | 0.47 | 2 | 34 | 4.61 |
| MI4r | 330.16 | 0.000006 | 0.390 | 0.47 | 2 | 20 | 4.20 |
| MI4n.3n | 330.31 | 0.000000 | 0.150 | 0.46 | 2 | 5 | 4.44 |
| MI4n.2r | 330.55 | 0.000167 | 0.232 | 0.47 | 2 | 110 | 5.15 |
| MI4n.2n | 330.75 | - | 0.206 | 0.47 | 1 | - | 6.13 |
| MI4n.1r | 330.87 | - | 0.119 | 0.47 | 1 | - | 6.82 |
| MI4n.1n | 330.97 | - | 0.094 | 0.48 | 1 | - | 5.60 |
| MI3r | 331.23 | - | 0.262 | 0.48 | 1 | - | 5.22 |
| MI3n.2n | 331.46 | 0.000005 | 0.231 | 0.50 | 2 | 19 | 6.46 |
| MI3n.1r | 331.50 | 0.000001 | 0.047 | 0.50 | 2 | 7 | 6.90 |
| MI3n.1n | 331.64 | 0.000002 | 0.135 | 0.51 | 2 | 11 | 5.48 |
| MI2r | 331.95 | 0.000082 | 0.313 | 0.53 | 2 | 77 | 5.23 |
| MI2n.3n | 332.08 | 0.000005 | 0.129 | 0.54 | 2 | 20 | 7.15 |
| MI2n.2r | 332.19 | 0.000013 | 0.103 | 0.54 | 2 | 30 | 9.14 |
| MI2n.2n | 332.25 | 0.000032 | 0.065 | 0.55 | 2 | 48 | 8.07 |
| MI2n.1r | 332.45 | - | 0.201 | 0.57 | 1 | - | 6.79 |
| MI2n.1n | 332.56 | - | 0.109 | 0.58 | 1 | - | 6.93 |
| MI1r.3r | 332.76 | - | 0.202 | 0.60 | 1 | - | 8.55 |
| MI1r.2n | 332.80 | - | 0.034 | 0.60 | 1 | - | 11.47 |
| MI1r.2r | 332.91 | - | 0.110 | 0.61 | 1 | - | 5.88 |
| MI1r.1n | 332.94 | - | 0.034 | 0.62 | 1 | - | 3.58 |

Supplementary Table 7. Late Cambrian-Upper Ordovician chron base ages, durations and optimisation statistics. Columns as in Supplementary Table 5. No $c_{95}$ values for pre-Moyero chrons relate to having onto two radiometric age points. Subchrons beginning a,b etc are considered tentative chrons based on single-sample data. For the LO1r.1n to UO1r interval reversal rates determined using a smoothing parameter (alpha) of 0.4, corresponding to an 8 chron bandwidth. For the mC1n- LO1r.ar interval these values are alpha=0.52, bandwidth=14 chrons. Base Ordovician estimated at 486.88 Myr and base Furongian at 491.91 Myr.

| Chron | Age (Myr) | $E_n$ (optimised height units) | Chron duration (Myr) | $c_{95}$ (Myr) | $N_i$ | $\sigma_T$ Myrs | Rev. rates (r per Myr) |
|---|---|---|---|---|---|---|---|
| UO1r | 454.73 | - | | 2.24 | 1 | - | 0.63 |
| UO1n.2n | 457.26 | - | 2.533 | 1.47 | 1 | - | 0.78 |
| UO1n.1r | 457.70 | 0.000006 | 0.440 | 1.37 | 2 | 0.117 | 0.99 |
| UO1n.1n | 458.26 | 0.000083 | 0.552 | 1.26 | 4 | 0.362 | 1.28 |
| MO2r.2r | 459.19 | 0.000087 | 0.937 | 1.10 | 3 | 0.395 | 1.24 |
| MO2r.1n | 459.68 | 0.000068 | 0.488 | 1.04 | 3 | 0.49 | 1.17 |
| MO2r | 461.21 | 0.000078 | 1.531 | 0.92 | 4 | 0.352 | 1.31 |
| MO2n.2n | 461.73 | 0.000059 | 0.519 | 0.91 | 4 | 0.305 | 1.66 |
| MO2n.1r | 462.11 | 0.000003 | 0.383 | 0.91 | 2 | 0.086 | 2.20 |
| MO2n.1n | 462.32 | - | 0.211 | 0.91 | 1 | - | 2.14 |
| MO1r.2r | 462.76 | - | 0.437 | 0.92 | 1 | - | 1.72 |
| MO1r.1n | 463.56 | 0.000021 | 0.795 | 0.97 | 2 | 0.466 | 1.61 |
| MO1r | 464.60 | 0.000555 | 1.046 | 1.09 | 3 | 0.112 | 1.81 |
| MO1n.2n | 464.72 | 0.000401 | 0.120 | 1.11 | 3 | 0.095 | 2.35 |
| MO1n.1r | 465.07 | 0.000421 | 0.352 | 1.16 | 4 | 0.092 | 2.93 |
| MO1n.1n | 465.27 | 0.000383 | 0.196 | 1.19 | 4 | 0.088 | 2.93 |
| LO1r.3r | 465.91 | 0.000023 | 0.638 | 1.31 | 2 | 0.026 | 2.67 |
| LO1r.2n | 465.97 | 0.000109 | 0.062 | 1.33 | 2 | 0.057 | 2.74 |
| LO1r.2r | 466.71 | 0.000011 | 0.737 | 1.49 | 2 | 0.018 | 2.87 |
| LO1r.1n | 466.76 | 0.000003 | 0.059 | 1.51 | 2 | 0.010 | 3.04 |
| LO1r.bn | Late Tremadocian- not estimated | | | | | | |
| LO1r.ar | 485.75 | - | | - | 1 | - | 4.23 |
| LO1r.an | 485.82 | - | 0.071 | - | 1 | - | 4.45 |
| LO1r | 485.90 | 0.00033 | 0.080 | - | 3 | 0.162 | 4.68 |
| LO1n.3n | 486.55 | - | 0.643 | - | 1 | - | 4.93 |
| LO1n.2r | 486.62 | - | 0.071 | - | 1 | - | 5.17 |
| LO1n.2n | 486.68 | 0.00025 | 0.061 | - | 3 | 0.143 | 5.45 |
| LO1n.1r | 486.88 | 0.00000 | 0.201 | - | 2 | 0.014 | 5.82 |
| LO1n.1n | 486.95 | - | 0.069 | - | 1 | - | 6.20 |
| FU4r | 487.17 | 0.00046 | 0.224 | - | 2 | 0.221 | 6.46 |
| FU4n.3n | 487.44 | 0.00040 | 0.265 | - | 3 | 0.180 | 6.50 |
| FU4n.2r | 487.55 | 0.00023 | 0.107 | - | 3 | 0.136 | 6.42 |
| FU4n.2n | 487.58 | 0.00023 | 0.037 | - | 3 | 0.134 | 6.12 |
| FU4n.1r | 487.84 | 0.00002 | 0.256 | - | 2 | 0.049 | 5.47 |
| FU4n.1n | 487.90 | 0.00003 | 0.062 | - | 2 | 0.056 | 4.58 |
| FU3r | 488.05 | - | 0.154 | - | 1 | - | 3.67 |
| FU3n | 488.33 | 0.00065 | 0.277 | - | 2 | 0.264 | 2.84 |
| FU2r | 488.79 | - | 0.456 | - | 1 | - | 2.22 |
| FU2n.ar | No estimated | - | - | - | - | - | |
| FU2n | 489.05 | - | 0.263 | - | 1 | - | 1.81 |
| FU1r.2r | 489.96 | - | 0.905 | - | 1 | - | 1.57 |
| FU1r.1n | 490.03 | - | 0.073 | - | 1 | - | 1.47 |
| FU1r.1r | 491.91 | - | 1.881 | - | 1 | - | 1.50 |
| FU1n | 492.20 | - | 0.292 | - | 1 | - | 1.73 |
| mC1r | 493.41 | - | 1.204 | - | 1 | - | 2.08 |
| mC1n.3n | 493.57 | - | 0.160 | - | 1 | - | 2.64 |
| mC1n.2r | 493.62 | - | 0.057 | - | 1 | - | 3.74 |
| mC1n.2n | 493.68 | - | 0.062 | - | 1 | - | 6.69 |
| mC1n.1r | 493.74 | - | 0.056 | - | 1 | - | - |

Supplementary Table 8. Runs of optimisation models for the Permian GPTS. Section names are the column tops (See Supplementary Table 1). The final used solution is the model P-4, derived from refining the previous models (starting with P-1), finally excluding the data from Tuyembetka. The Sukhona section was not excluded, since its one of the only two sections which displays the transition across LP0r-LP1n, which is not interrupted by hiatus or lack of sampling. Superscripts T (transgressive) and R (regressive) on $D$j values indicate the rate functions used. No subscripts on $D_j$ indicate constant rate function used. ST (section tops) and SB (section bottoms) superscripts on $T_p$ values are constraints at their limits in the optimised model. Constraints applied for all 4 models: constraints using section top: Tetyushi (<LP0r), Sukhona (<LP2n.3n), Nammal (<LT1n), Abadeh (<LT1n), Taiyuan (<LP1r); constraints using section bottom: Pizhma (>GU3n), Linsui (>LP0r), Abadeh (>LP2n.1n); Nammal- hiatus in bed 27 >LP0r; mid-section (data gap) constraints: Boyevaya $N_2P$ > LP1n; Shangsi (bed 7) top N <LP0r. For each model the three chrons with the largest $E_n$ values are also shown. Flagged in yellow are the two largest $D_j$ and smallest $T_p$ (<=0.05), which indicate section data with the most anomalous statistics. The degrees of freedom (DOF) for the 4 models are from model P-1, 41, 43, 46 and 41 respectively, where DOF= variables and constants at limits minus the 2 equality constraints that set the scale at 0 and 1.0. The total number of magnetozone boundaries in model P-4 is 116, and in the other models is 122.

| **Model**, parameters | Khei-yaga | Murygino | Monastyrki | Boyevaya | Tuyembetka | Sambullak | Tetyushi | Cherumuska | Sukhona | Pizhma | Oparino | W.Texas | Linsui | Wulong | Shangsi | Taiyuan | Nammal | Abadeh |
|---|---|---|---|---|---|---|---|---|---|---|---|---|---|---|---|---|---|---|
| **P-1**, $E_{tot}$=0.00341, $D_s$=0.1930, LP0r.1n.$E_n$=0.00028, LP0r.2r.$E_n$=0.00032, LP1n.1n.$E_n$=0.00043 | | | | | | | | | | | | | | | | | | |
| $D_j$ | 0.207 | $0.137^{T}$ | 0.118 | 0.100 | 0.353 | 0.249 | 0.100 | 0.057 | 0.296 | 0.194 | 0.370 | 0.186 | $0.234^{T}$ | $0.179^{R}$ | 0.165 | 0.105 | 0.263 | 0.160 |
| $T_p$ | 0.55 | 0.90 | 0.47 | 0.01 | 0.48 | 0.70 | 0.96 | 0.66 | $0.79^{ST}$ | 0.67 | 0.91 | 0.93 | $0.24_{SB}$ | 0.49 | 0.56 | $0.65^{ST}$ | 0.84 | $0.88_{SB}$ |
| **P-2,** $E_{tot}$=0.00322, $D_s$=0.1759, LP0r.1n. $E_n$ =0.00027, LP0r.2r. $E_n$ =0.00032, LP1n.1n. $E_n$ =0.00038 | | | | | | | | | | | | | | | | | | |
| $D_j$ | 0.181 | $0.132^{T}$ | 0.108 | 0.081 | 0.342 | 0.199 | 0.100 | 0.051 | 0.350 | 0.177 | $0.171^{T}$ | 0.202 | $0.221^{T}$ | $0.178^{R}$ | 0.160 | 0.105 | 0.256 | 0.151 |
| $T_p$ | 0.99 | 0.91 | 0.80 | 0.05 | 0.59 | 0.84 | 0.92 | 0.50 | $0.61^{ST}$ | $0.80^{SB}$ | 0.96 | 0.95 | $0.29^{SB}$ | 0.51 | 0.54 | $0.67^{ST}$ | 0.83 | 0.83 |
| **P-3,** $E_{tot}$=0.00352, $D_s$=0.1905, LP0r. $E_n$ =0.00035, LP0r.2r. $E_n$ =0.00032, LP1n.1n. $E_n$ =0.00033 | | | | | | | | | | | | | | | | | | |
| $D_j$ | 0.199 | $0.137^{T}$ | 0.106 | $0.237^{T}$ | 0.374 | 0.248 | 0.101 | 0.061 | 0.342 | 0.247 | $0.118^{T}$ | 0.175 | $0.267^{T}$ | $0.168^{R}$ | 0.162 | 0.098 | 0.213 | 0.176 |
| $T_p$ | 0.84 | 0.90 | 0.48 | 0.71 | 0.59 | 0.82 | 0.72 | 0.70 | $0.28^{ST}$ | $0.90^{SB}$ | 0.67 | 0.74 | $0.38^{SB}$ | 0.49 | 0.82 | $0.32^{ST}$ | 0.94 | 0.25 |
| **P-4**, $E_{tot}$=0.00310, $D_s$=0.1685, LP0r.2r. $E_n$ =0.00032, LP1n.1n. $E_n$ =0.00029, LP2n.1r. $E_n$ =0.00030 | | | | | | | | | | | | | | | | | | |
| $D_j$ | 0.189 | $0.133^{T}$ | 0.104 | $0.043^{T}$ | - | 0.238 | 0.102 | 0.054 | 0.420 | 0.226 | $0.128^{T}$ | 0.212 | $0.266^{T}$ | $0.167^{R}$ | 0.178 | 0.040 | 0.228 | 0.136 |
| $T_p$ | 0.89 | 0.94 | 0.76 | 0.46 | - | 0.69 | 0.75 | 0.78 | $0.49^{ST}$ | $0.99^{SB}$ | 0.73 | 0.76 | $0.40^{SB}$ | 0.49 | 0.47 | $0.39^{ST}$ | 0.78 | 0.84 |

Supplementary Table 9. Optimisation model for the Carboniferous GPTS. Section names are the column tops (See Supplementary Figure 1). Subscripts T (transgressive) and R (regressive) on $D$j values indicate the rate functions used. No superscripts on $D_j$ indicate linear rate function used. ST (section tops) and SB (section bottoms) superscripts on $T_p$ values are constraints at their limits in the optimised model. Constraints applied: constraints using section top: Minudie <MI7n.3n; L-Pottsville <MI5r; L-Lavelle <M3r; constraints using section base: Maringouin >MI4n.2n, L-Pottsville >MI2n.1r; No mid section constraints. The three chrons with the largest $E_{tot}$ values are also shown. Flagged in yellow is the largest $D_j$ which indicate section data with the most anomalous statistics. The degrees of freedom for the model is 13, and the total number of magnetozone boundaries in the model is 49.

| **Model**, parameters | Minudie | Maringouin | U-Pottsville | L-Pottsville | U-Lavelle | L-Lavelle |
|---|---|---|---|---|---|---|
| **Carb-1**, $E_{tot}$=0.00053, $D_s$=0.0669, MI4r.$E_n$=0.000167, MI2n.2n.$E_n$=0.00098, MI2r.$E_n$=0.00082 | | | | | | |
| $D_j$ | 0.054 | 0.052 | 0.071 | 0.047 | 0.067 | 0.111 |
| $T_p$ | $0.53^{ST}$ | $0.92^{SB}$ | 0.97 | $0.73^{SB}$ | 0.64 | 0.75 |

Supplementary Table 10. Mid-late Ordovician GPTS optimisation model. Several other improvements were attempted, but failed, firstly using a regressive rate model for Mójcza, but the statistics were slightly worse than model O-1. Similarly a transgressive model for Kudrino was attempted, by it produced very similar statistics to model O-1, so it was not a significant improvement. Subscripts T (transgressive) and R (regressive) on $D$j values indicate the rate functions used. No superscripts on $D_j$ indicate linear rate function used. ST (section tops) and SB (section bottoms) superscripts on $T_p$ values are constraints at their limits in the optimised model. Constraints applied: constraints using section top: L-Rozkhova <MO2n, Kudrino <UO1n.1r; constraints using section base: U. Gullhögen >MO1r.2r, Kudrino >MO1r, Alexeeva >MO2r; Constraints mid section: U Gullhögen base Dalby-N >UO1n.2n. The three chrons with the largest $E_{tot}$ values are also shown. Flagged in yellow are the two largest $D_j$ which indicate section data with the most anomalous statistics. The degrees of freedom for the model is 18, and the total number of magnetozone boundaries in the model is 46.

| **Model**, parameters | Mojcza | L-Gullhogen | U-Gullhogen | U-Rozkhova | l-Rozkhova | Moyero | Kudrino | Polovinka | Alexeeva |
|---|---|---|---|---|---|---|---|---|---|
| **O-1**, $E_{tot}$=0.00231, $D_s$=0.1965, MO1n.1r.$E_n$=0.00042, MO1n.2n.$E_n$=0.00040, MO1r.$E_n$=0.00056 | | | | | | | | | |
| $D_j$ | 0.352 | 0.175 | 0.250 | 0.080 | 0.090 | 0.192 | 0.188 | 0.070 | 0.372 |
| $T_p$ | 0.98 | 0.96 | $1.00^{SB}$ | 0.73 | 0.71 | 0.83 | 0.39 | 0.65 | $0.80^{SB}$ |

Supplementary Table 11. Optimisation GPTS model for the late Cambrian-Lower Ordovician. Section names are the column tops (See Supplementary Figure 2). The all linear rate model is only slightly poorer ($D_s$=0.282, $E_{tot}$=0.00262). Other types of rate models for Kulymbe all gave poorer models, and changing the top-most normal magnetozone at Kulymbe to correspond to LO1n, also gave a poorer model. The large $D_s$ value of Kulyumbe relates to the large difference in relative thickness of FU4n.3n and FU4n.1r- FU4n.3n interval compared to the Black Mountain section data. Subscripts T (transgressive) and R (regressive) on $D_j$ values indicate the rate functions used. No superscripts on $D_j$ indicate linear rate function used. ST (section tops) and SB (section bottoms) superscripts on $T_p$ values are constraints at their limits in the optimised model. Constraints applied: using section tops: Black Mountain <LO1r.an; constraints using section base: Black Mountain >FU1r.2r, Zhaogehuang >FU4n.1r; No mid section constraints. The two chrons with the largest $E_{tot}$ values are also shown. Flagged in yellow is the largest $D_j$ which indicate section data with the most anomalous statistics. The degrees of freedom for the model is 7, and the total number of magnetozone boundaries in the model is 25.

| **Model**, parameters | Zhaogezhuang | Black Mountain | Kulyumbe |
|---|---|---|---|
| **CO-1**, $E_{tot}$=0.00260, $D_s$=0.280, FU3n.$E_n$=0.00065, FU4r.$E_n$=0.00046 | | | |
| $D_j$ | 0.137$^{T}$ | 0.147 | 0.556 |
| $T_p$ | 0.41$^{SB}$ | 0.96$^{SB}$ | 0.37 |

# Supplementary Note 1

## Data selection and quality issues

Magnetostratigraphic study quality criteria have been proposed[100], consisting of 10 quality criteria (1 to 11, excluding #5, listed below), based around the conventional paleopole-type quality criteria. An additional criteria #5 has been added here, since sometimes sampling density is insufficient to define magnetozones adequately. Those marked with a cross (x) are generally satisfied in the data used here.

**Sampling and other data:**

1) Sufficient fossil age control to stage or better (x)
2) Radiometric dates in the section
3) Multiple sections studied (repeatable patterns shown) (x)
4) Data shown in depth (m) in section (with log) (x)
5) Sufficient sampling density with respect to chron durations (x)
6) Numerical data published as Dec/Inc or VGP latitude (x)

**Experimental techniques/analysis/magnetisation directions**

7) Complete stepwise demagnetisation (x)
8) PCA fitting methods (x)
9) Magnetic mineralogy determined
10) Positive field tests for age of magnetisation
11) Antipodal directions (x)

In the composites produced here, generally the number of magnetostratigraphic studies for particular age intervals is small (as opposed to palaeopole type studies, where the opposite is true), so using these criteria for data elimination in a small dataset is not helpful.

The data used here in constructing the composites are self selecting, since the compositing procedures generally requires criteria 1,2,3,4,5,6 and 11. Criteria#3 is here since, by the nature of the compositing procedures, one requires data from several sections to be repeatable, even if they don't come from a single study. All of the studies used here post-date the early 199O's, when all usable magnetostratigraphic studies typically have criteria 7 and 8 (even for the Permian Tatarian datasets).

Criteria #1 and #5 are not yes/no type flags, but rather gradational in character, so involve a good degree of subjective judgement, and can only be utilised into a magnetostratigraphy-like Q-factor, if they are scored say 1 to 3 (a significant issue, which has not been addressed in previous work). Clearly Criteria #2 would be important for a study like this, but in reality few magnetic polarity data can be directly related to radiometric data in the same section, and as here often the best to expect is a correlation to the magnetostratigraphy by some independent means. Criteria #10 is difficult to utilise in magnetostratigraphic studies, since often the most detailed/best quality polarity results come from flat lying units, without hiatus, so applying fold and conglomerate tests is not common in magnetostratigraphic studies.

For these reasons filtering data according to quality-criteria data out does not guarantee a better a GPTS-e.g. you could loose validated chrons seen in more than one study. The compositing procedures used here, also needs to use the largest amount of data, with high sampling density, reasonable biostratigraphy, some tie to radiometric dates and the largest stratigraphic coverage in order to function effectively.

## Implementation in Excel

**Transgressive rate functions**, simulating smoothly increasing sedimentation rates are:

$\mathbf{T}_{j,i} = \beta_j + \alpha_j * F_j(\mathbf{H}_{j,i}; \lambda_j)$, where F(x; λ) is the cumulative exponential distribution function with parameter λ ≥0, implemented in Excel as EXPONDIST(x, λ, TRUE).

**Regressive rate functions,** simulating smoothly decreasing sedimentation rates are:

$\mathbf{T}_{j,i} = \beta_j + \alpha_j / F_j(\mathbf{H}_{j,i}; \lambda_j)$, where F(x; λ) is the exponential distribution probability density function with λ ≥0 and where $\alpha_j > 0$), implemented Excel as EXPONDIST(x, λ, FALSE).

These notes relate to Excel 2007, more recent versions have a different Solver interface, which has not been used here. Generally either Newton or Conjugate search's (Supplementary Figure 8) provided convergence on the final solution, but near the convergence point switching between these two options generally reached the convergence point better. Sometimes more than 50 seconds and/or 1000 iterations were needed to reach convergence for some solutions.

For large datasets, convergence was sometimes slow and Solver may fail to fix a badly fitting section. A plot of $\mathbf{T}_{j,i}$ against $\mathbf{P}_i$ greatly helps in a visual assessment of the fit, like in Supplementary Figures 4a,c. If the start values of $\mathbf{T}_{j,i}$ are far from a fair visual match to the optimised composite, trials indicate it is best changing the $\alpha_j$, $\beta_j$ and $\lambda_j$ values manually (prior to optimisation) to approach something that looks reasonable, otherwise Solver may fail to find a sensible solution. Solution-finding can also be reached by performing the optimisation initially in smaller groups of section data, followed by the whole dataset. For a large dataset, this seems to find a more rapid solution. Construction of an initial correlation like Supplementary Figures 1 & 2 helps in this process also. Upper and lower scale values of approximately 0 and 1 seem to make the Solver optimisation process more stable, and enable Solver to find a final stable solution easier. Different values for the upper and lower scale limits could be used, but did not work effectively in Solver. The Permian and mid Ordovician datasets used are shown in the accompanying Excel file, with additional notes on how to implement the method in the spreadsheet, explained on the Ordovician sheet.

# Supplementary References


1. Opdyke, N.D., Giles, P.S. & Utting, J. Magnetic polarity stratigraphy and palynostratigraphy of the Mississippian-Pennsylvanian boundary interval in eastern North America and the age of the beginning of the Kiaman. *Geol. Soc. Am. Bull.* **126**, 1068-1083 (2014).

2. Hounslow, M. W., Davydov, V. I., Klootwijk, C. T., & Turner, P. Magnetostratigraphy of the Carboniferous: a review and future prospects. *Newsletter on Carboniferous Stratigraphy*, **22**, 35-41 (2004).

3. Kouchinsky A. *et al.* The SPICE carbon isotope excursion in Siberia: a combined study of the upper Middle Cambrian–lowermost Ordovician Kulyumbe River section, northwestern Siberian Platform. *Geol. Mag*. **145**, 609–622 (2008).

4. Terfelt, F., Eriksson, M. E., & Schmitz, B. The Cambrian–Ordovician transition in dysoxic facies in Baltica—diverse faunas and carbon isotope anomalies. *Palaeogeography, Palaeoclimatology, Palaeoecology*, **394**, 59-73 (2014).

5. Cooper, R. A., Nowlan, G. S., & Williams, S. H. Global stratotype section and point for base of the Ordovician System. *Episodes*, **24**, 19-28 (2001).

6. Peng, S. et al. M. Global Standard Stratotype-section and Point (GSSP) of the Furongian Series and Paibian Stage (Cambrian). *Lethaia*, **37**, 365-379 (2004).

7. Peng, S. *et al.* Global standard stratotype-section and point (GSSP) for the base of the Jiangshanian Stage (Cambrian: Furongian) at Duibian, Jiangshan, Zhejiang, southeast China. *Episodes*, **35**, 462-477 (2012).

8. Landing, E., Westrop, S. R. & Adrain, J. M. The Lawsonian Stage–the Eoconodontus notchpeakensis (Miller, 1969) FAD and HERB carbon isotope excursion define a globally correlatable terminal Cambrian stage. *Bulletin of Geosciences*, **86**, 621-640 (2011).

9. Ripperdan, R. L., Magaritz, M. & Kirschvink, J. L. Carbon isotope and magnetic polarity evidence for non-depositional events within the Cambrian-Ordovician boundary section near Dayangcha, Jilin Province, China. *Geological Magazine*, 130, 443-452 (1993).

10. Agterberg, F.P. in *A geologic time scale* (eds. Gradstein, F., Ogg, J. & Smith, A.). 106-126 (Cambridge University Press, 2004).

11. Kim, C. & Storer, B.E. Reference values for Cook's distance. *Communications in Statistics - Simulation and Computation* **25**, 691-708 (1996).

12. Ogg, J. G. in *The Geologic Time Scale 2012*. (eds Gradstein, F. M., Ogg, J. G., Schmitz, M. D. et al.) 85-114 (Elsevier Publ. 2012).

13. Hounslow, M.W. & Muttoni, G. in *The Triassic Timescale*, Special Publication of the Geological Society, 334 (ed Lucas, S.G. ) 61-102 (Geological Society, London, 2010).

14. Iosifidi, A. G. & Khramov, A. N. Magnetostratigraphy of Upper Permian Sediments in the Southwestern Slope of Pai-Khoi (Khei-Yaga River Section): Evidence for the Global Permian–Triassic Crisis. *Izvestiya Physics of the Solid Earth*, **45**, 3–13 (2009).

15. Biakov, A.S. A New Permian Bivalve Zonal Scale of Northeastern Asia. Article 2: Correlation Problems. *Russian Journal of Pacific Geology* 7, 1–15 (2013).

16. Burov, B.V., Zharkov, I.Y., Nurgaliev, D.K., Balabonov, Y.P., Borisov, A.S. & Yasonov, P.G. in *Stratotypes and reference sections of the Upper Permian in the region of the Volga and Kama Rivers* (eds. Esaulova, N.K., Lozovsky, V.R. & Rozanov, A.Y. ) 236-270 (GEOS, 1998).

17. Kotlyar, G.V & Pronina-Nestell, G.P. Report of the committee on the Permian system of Russian. *Permophiles* **46**, 9-13 (2005).

18. Taylor, G.K., *et al.* Magnetostratigraphy of Permian/Triassic boundary sequences in the Cis-Urals, Russia: no evidence for a major temporal hiatus. *Earth and Planetary Science Letters* **281**, 36-47 (2009).

19. Lozovsky, V. R. Chapter 7: The Permian- Triassic boundary. in *Stratotypes and reference sections of the Upper Permian in the region of the Volga and Kama Rivers* (eds. Esaulova, N.K., Lozovsky, V.R. & Rozanov, A.Y.) 271-281 (GEOS, 1998).

20. Lozovsky, V. R. Minikh, M. G., Grunt, T. A. Kukhtinov, D. A. Ponomarenko A. G. & Sukacheva, I. D. The Ufimian Stage of the East European Scale: Status, Validity, and Correlation Potential. *Stratigraphy and Geological Correlation* **17**, 602–614 (2009).

21. Newell, A.J. *et al.* Disruption of playa–lacustrine depositional systems at the Permo-Triassic boundary: evidence from Vyazniki and Gorokhovets on the Russian Platform. *Jour. Geol. Soc. London* **167**, 695–716 (2010).

22. Khramov, A.N., Komissarova, R.A., Iosifidi, A.G. Popov, V.V. & Bazhenov, M.L. 2006. in *Problems of Geocosmos* (eds. Troyan, V.N., Semenov, V.S., Kubyshkina, M.V. ) 317–321, (6th International Conference St. Petersburg State University, St. Petersburg, 2006).

23. Gialanella1, P.R. *et al.* Late Permian magnetostratigraphy on the eastern Russian platform. *Geologie en Mijnbouw* **76,** 145–154, (1997).

24. Westfahl , M., Surkis, Y.F., Gurevich, E.L. & Khramov, A.N. Kiama-Illawarra geomagnetic reversal recorded in the Tatarian Stratotype (the Kazan region). *Izvestiya, Physics of the solid Earth* **41**, 634-653 (2005).

25. Steiner, M.B. (2006). in *Non-Marine Permian Biostratigraphy and Biochronology*. Geological Society, London, Special Publications, **265**, (eds Lucas, S. G., Cassinis, G. & Schneider, J. W.) 15-38 (2006).

26. Bowring, S.A., Erwin, D.H., Jin, Y.G. Martin, M.W., Davidek, K. & Wang, W. U-Pb Zircon geochronology and Tempo of the End-Permian Mass extinction. *Science* **280**, 1039-1045 (1998).

27. Lambert, L.L. Wardlaw, B.R. & Henderson, C.H. *Mesogondolella* and *Jinogondolella* (Conodonta): Multielement definition of the taxa that bracket the basal Guadalupian (Middle Permian Series) GSSP. *Palaeoworld* **16**, 208-221 (2007).

28. Rush, J. & Kerans, C. Stratigraphic response across a structurally dynamic shelf: the latest Guadalupian composite sequence at Walnut Canyon, New Mexico, USA . *Jour. Sedim. Res.* **80**, 808–828 (2010).

29. Barnaby, R.J. & Ward, W.B. Outcrop analog for mixed siliciclastic–carbonate ramp reservoirs—stratigraphic hierarchy, facies architecture, and geologic heterogeneity: Grayburg Formation, Permian basin, USA. *Jour. Sedim. Res.* **77**, 34–58 (2007).

30. Li, H. & Wang, J. Magnetostratigraphy of the Permo-Triassic boundary section of Meishan of Changxing, Zhejang. *Science in China* **8**, 652-658 (1989).

31. Liu, Y.Y., Zhu, Y.M. & Tian, W.H. New magnetostratigraphic results from the Meishan section, Changxing County, Zheijiang, China. *Earth Science Journal of China University of Geosciences* **24**, 151-154 (1999).

32. Meng, X., Hu, C., Wang, W., Liu, H. Magnetostratigraphic Study of Meishan Permian-Triassic Section, Changxing, Zhejiang Province, China. *Journal of China University of Geosciences* **11**, 361-365 (2000).

33. Mundil, R., Ludwig, K.R., Metcalfe, I. & Renne, P.R. Age and timing of the Permian mass extinctions: U/Pb dating of closed-system zircons. *Science* **305**, 1760-1763 (2004).

34. Mundil, R., Palfy, J. Renne, P.R. & Brack, P. in *The Triassic Timescale* Special Publication of the Geological Society, **334** (ed. Lucas, S.G.) 41-60, (Geological Society, London, 2010).

35. Shen, S-Z., Henderson, C.M., Bowring, S.A., Cao, C-Q., Wang, Y., Zhang, H., Zhang, Y-C., Mu, L. High resolution Lopingian (late Permian) timescale of South China. *Geological Journal* **45**, 122-134 (2010).

36. Shen S-Z. et al. Calibrating the end-Permian mass extinction. *Science* **334**, 1367–1372 (2011).

37. Jin, Y.G., Wang, Y., Henderson, C., Wardlaw, B.R., Shen, S. & Cao, C. The global boundary stratotype section and point (GSSP) for the base of the Changhsingian Stage (Upper Permian). *Episodes* **29**, 175-182 (2006).

38. Yuan, D-X., Shen, S-Z., Henderson, C.M., Chen J., Zhang, H. & Feng, H-Z. Revised conodont-based integrated high-resolution timescale for the Changhsingian Stage and end-Permian extinction interval at the Meishan sections, South China. *Lithos* **204**, 220–245 (2014).

39. Heller, F., Chen, H., Dobson, J. & Haag, M. Permian-Triassic magnetostratigraphy – new results from South China. *Earth and Planetary Science Letters* **89**, 281-295 (1995).

40. Chen, Z-Q., Campi, M.J., Shi, G.R., & Kaiho, K. Post extinction brachiopod faunas from the Late Permian Wuchiapingian coal series of South China. *Acta Palaeontol. Pol.* **50**, 343-363 (2005).

41. Ali, J.R., Thompson, G.M., Song, X. & Wang, Y. Emeishan basalts (SW China) and the end-Gudalupian crisis: magnetobiostratigraphic constraints. *Jour. Geol. Soc. London*. **159**, 21-29 (2002).

42. Zheng, L. Yang, Z. Tong, Y. Yuan, W. Magnetostratigraphic constraints on two-stage eruptions of the Emeishan continental flood basalts. *Geochemistry Geophysics Geosystems* **11**, doi:10.1029/2010GC003267 (2010).

43. Liu, C., Pan, Y., Zhu, R. New paleomagnetic investigations of the Emeishan basalts in NE Yunnan, southwestern China: Constraints on eruption history. *Jour. Asian Earth Sci.* **52**, 88-97 (2012).

44. Zhong Y-T, He B. Mundil R. & Xu, Y-G. CA-TIMS zircon U–Pb dating of felsic ignimbrite from the Binchuan section: Implications for the termination age of Emeishan large igneous province. *Lithos* **204**, 14–19 (2014).

45. He, B. *et al.* Age and duration of the Emeishan flood volcanism, SW China: Geochemistry and SHRIMP zircon U–Pb dating of silicic ignimbrites, post-volcanic Xuanwei Formation and clay tuff at the Chaotian section. *Earth and Planetary Science Letters* **255,** 306–323 (2007).

46. Sun, Y. *et al.* Dating the onset and nature of the Middle Permian Emeishan large igneous province eruptions in SW China using conodont biostratigraphy and its bearing on mantle plume uplift models. *Lithos* **119**, 20-33 (2010).

47. Chen, H-H., Sun, S. & Li, J-L. Permo Triassic magnetostratigraphy in Wulong area, Sichuan, China. *Science in China (series B)* **37**, 203-212 (1994).

48. Jin, Y.G., Shang, Q.H. & Cao C.Q. Late Permian magnetostratigraphy and its global correlation. *Chinese Science Bulletin* **45**, 698-704 (2000).

49. Heller, F., Lowrie, W., Huanmei, L. & Junda, W. Magnetostratigraphy of the Permo-Triassic boundary section at Shangsi (Guangyuan, Sichuan Province, China). *Earth Planet. Sci. Lett.* **88**, 348-356 (1988).

50. Steiner, M.B., Ogg, J., Zhang, Z. & Sun, S. The Late Permian/early Triassic magnetic polarity time scale and plate motions of south China. *Jour. Geophys. Res.* **94**, 7343-7363 (1989).

51. Glen, J.M., Nomade, S., Lyons, J.L, Metcalfe, I., Mundil, R. & Renne, P.R. Magnetostratigraphic correlations of Permian-Triassic marine and terrestrial sediments from western China. *Jour. Asian Earth Sci.* **36**, 521–540 (2009).

52. Lai, X., Yang, F., Hallam, A. & Wignall, P.B. in *The Paleozoic - Mesozoic Boundary* (ed. Yin, H.F.) 113-124 (China University of Geosciences Press, 1996).

53. Sun, Y., Lai, X., Jiang, H., Luo, G., Sun, S., Yan, C. & Wignall, P.B. Guadalupian (Middle Permian) conodont Faunas at Shangsi section, Northeast Sichuan Province. *Jour. China Univ. of Geosci.* **19**, 451-460 (2008).

54. Embleton, B.J.J., McElhinny, M.W., Zhang, Z. & Li, Z.X. Permo-Triassic magnetostratigraphy in China: the type section near Taiyuan, Shanxi Province, North China .*Geophys. J. Int.* **126**, 382–388 (1996).

55. Menning, M. & Jin, Y. Comment on 'Permo-Triassic magnetostratigraphy in China: the type section near Taiyuan, Shanxi Province, North China' by B.J.J. Embleton, M.W. McElhinny, X. Ma, Z. Zhang and Z.X. Li. *Geophys. J. Int.* **133**, 213–216 (1998).

56. Stevens L.G., Hilton, J., Bond, D.P.G., Glasspool, I.J. & Jardine, P.E. Radiation and extinction patterns in Permian floras from North China as indicators for environmental and climate change. *Jour. Geol. Soc. London* **168**, 607–619 (2011).

57. Haag, M. & Heller, F. Late Permian to Early Triassic magnetostratigraphy. *Earth Planet. Sci. Lett.* **107**, 42–54 (1991).

58. Wardlaw, B.R. & Pogue, K.R. in *The Permian of Northern Pangea 2, Sedimentary Basins and Economic Resources* (eds Scholle, P.A., Peryt, T.M., & Ulmer-Scholle, D.S. ) 215-224 (Springer-Verlag, 1995).

59. Wardlaw, B.R. & Mei, S. in *Proceedings of the international conference on Pangea and the Palaeozoic-Mesozoic transition* (eds Yin, H. & Tong, J.) 154-156, (Wuhan China, China Univ. of Geosciences Press. 1999)

60. Waterhouse, J.B. Lopingian (Late Permian) stratigraphy of the Salt Range, Pakistan and Himalayan region. *Geol. J.* **45**, 264–284 (2010).

61. Baud, A., Atudorei V. & Sharp, Z. The Upper Permian of the Salt Range area revisted: new stable isotope data. *Permophiles*, **27**, 39-41 (1995).

62. Mertmann, D. Evolution of the marine Permian carbonate platform in the Salt Range (Pakistan). *Palaeogeography, Palaeoclimatology, Palaeoecology* **191**, 373-384 (2003).

63. Gallet, Y., Krystyn, L., Besse, J., Saidi, A. & Ricou, L-E. New constraints on the upper Permian and Lower Triassic geomagnetic polarity timescale from the Abadeh section (central Iran). *Jour. Geophys. Res.* **105**, 2805-2815 (2000).

64. Shen, S-Z. & Mei, S.H. Lopingian (Late Permian) high-resolution conodont biostratigraphy in Iran with comparison to South China zonation. *Geol. J.* **45**, 135–161 (2010).

65. Mei, S. & Henderson, C.M. Comments on some Permian conodont faunas reported from SE Asia and adjacent areas and their global correlation. *Jour. Asian Earth Sci.* **20**, 599-608 (2002).

66. Opdyke, N.D. & Di Venere, V.J. The Magnetic Polarity Stratigraphy of the Mauch Chunk Formation, Pennsylvania *Proceedings of the National Academy of Sciences of the United States of America*, **101**, 13423-13427 (2004).

67. DiVenere, V. J., & Opdyke, N. D. Magnetic polarity stratigraphy and Carboniferous paleopole positions from the Joggins section, Cumberland structural basin, Nova Scotia. *Journal of Geophysical Research: Solid Earth*, **96**, 4051-4064 (1991).

68. Cózar, P. & Somerville, I. D. Problems correlating the late Brigantian–Arnsbergian Western European substages within northern England. *Geological Journal*. DOI: 10.1002/gj.2700 (2016).

69. DiVenere, V. J. & Opdyke, N. D. Paleomagnetism of the Maringouin and Shepody formations, New Brunswick: a Namurian magnetic stratigraphy. *Canadian Journal of Earth Sciences*, **27**, 803-810 (1990).

70. Schätz M. *et al*. Paleomagnetism of Ordovician carbonate rocks from Malopolska Massif, Holy Cross Mountains, SE Poland — Magnetostratigraphic and geotectonic implications. *Earth Planet. Sci. Lett*. **244**, 349–360 (2006).

71. Trela, W. Condensation and phosphatization of the Middle and Upper Ordovician limestones on the Malopolska Block (Poland): Response to paleoceanographic conditions. *Sedimentary Geology* **178**, 219–236 (2005).

72. Dzik, J. & Pisera A. Ordovician carbonate platform ecosystem of the Holy Cross Mountains. *Palaeontologia Polonica* **53**, 5-41 (1994).

73. Torsvik, T.H., Trench, A., Lobmann, K.C. & Dunn, S. Lower Ordovician reversal asymmetry: An artefact of remagnetization or nondipole field disturbance? *Jour. Geophys. Res*. **100**, 17885-17898 (1995).

74. Holmer, L.E. Middle Ordovician phosphatic inarticulate brachiopods from Vastergötland and Dalarna, Sweden. *Fossils and Strata*, **26**, 1-172 (1989).

75. Bergström S.M. & Löfgren, A. The base of the global Dapingian Stage (Ordovician) in Baltoscandia: conodonts, graptolites and unconformities. *Earth and Environmental Science Transactions of the Royal Society of Edinburgh* **99**, 189–212 (2009).

76. Mellgren J.I.S. & Eriksson, M.E. Untangling a Darriwilian (Middle Ordovician) palaeoecological event in Baltoscandia: conodont faunal changes across the 'Täljsten' interval. *Earth and Environmental Science Transactions of the Royal Society of Edinburgh* **100**, 353–370 (2010).

77. Lindskog, A. Palaeoenvironmental significance of cool-water microbialites in the Darriwilian (Middle Ordovician) of Sweden. *Lethaia* **47**, 187–204 (2014).

78. Ainsaar L., Kaljo, D., Martma, T., Meidla, Y., Männik, P., Nõlvak, J. & Tinn, O. Middle and Upper Ordovician carbon isotope chemostratigraphy in Baltoscandia: A correlation standard and clues to environmental history. *Palaeogeography Palaeoclimatology Palaeoecology* **294**, 189–201 (2010).

78. Pavlov, V.E., Rodionov, V.P., Khramov, A.N. & Gallet, Y. Magnetostratigraphy of the Polovinka key section, midstream Lena River: did the geomagnetic polarity change in the early LLandeilian? *Izvestiya, Physics of the Solid Earth* **35,** 402-412 (1999).

80. Sennikov N.V., Tolmacheva T.Yu., Obut O.T., Izokh N.G. & Lykova E.V. Zonation of the Siberian Ordovician deposits based on pelagic groups of fauna. *Russian Geology and Geophysics* **56**, 594–610 (2015).

81. Bergström, S.M., Chen, X., Gutierrez-Marco, J.C. & Dronov, A. The new chronostratigraphic classification of the Ordovician System and its relations to major regional series and stages and to $\delta^{13}C$ chemostratigraphy. *Lethaia* **42**, 97–107 (2009).

82. Pavlov, V.E., Veselovskiy R.V., Shatsillo, A. V. & Gallet, Y. Magnetostratigraphy of the Ordovician Angara/Rozhkova River Section: Further Evidence for the Moyero Reversed Superchron. *Izvestiya Physics of the Solid Earth* **48,** 297–305 (2012).

83. Pavlov, V.E., Bachtadse, V. & Mikhailov, V. New Middle Cambrian and Middle Ordovician palaeomagnetic data from Siberia: Llandelian magnetostratigraphy and relative rotation between the Aldan and Anabar–Angara blocks. *Earth Planet. Sci. Lett.* **276**, 229–242 (2008).

84. Pavlov, V., & Gallet, Y. Upper Cambrian to Middle Ordovician magnetostratigraphy from the Kulumbe river section (northwestern Siberia). *Physics of the Earth and Planetary Interiors*, **108**, 49-59 (1998).

85. Ripperdan, R. L. & Kirschvink, J. L. 1992. in *Global Perspectives on Ordovician Geology* (eds Webby, B. D. & Laurie, J. R.), 381–94 (Rotterdam, A. A. Balkema).

86. Yang, Z., Otofuji, Y., Sun, Z. & Huang, B. Magnetostratigraphic constraints on the Gondwanan origin of North China: Cambrian/Ordovician boundary results. *Geophysical Journal International* **151**, 1–10 (2002).

87. Burgess S.D., Bowring S., & Shen, S-Z. High-precision timeline for Earth's most severe extinction. *PNAS* **111**,  3316–3321 (2014).

88. Schmitz, M.D. in *The Geologic Time Scale, Vol I.* (eds Gradstein, F. M., Ogg, J. G., Schmitz, M. & Ogg, G.) 115-126 (Elsevier, 2012).

89. Pointon, M. A., Chew, D. M., Ovtcharova, M., Sevastopulo, G. D. & Crowley, Q. G. New high-precision U–Pb dates from western European Carboniferous tuffs; implications for time scale calibration, the periodicity of late Carboniferous cycles and stratigraphical correlation. *Journal of the Geological Society*, **169**, 713-721 (2012).

90. Gastaldo, R. A., Purkyňová, E., Šimůnek, Z. & Schmitz, M. D. Ecological persistence in the Late Mississippian (Serpukhovian, Namurian A) megafloral record of the Upper Silesian Basin, Czech Republic. *Palaios*, **24**, 336-350 (2009).

91. Davydov, V. I., Crowley, J. L., Schmitz, M. D. & Poletaev, V. I. High-precision U-Pb zircon age calibration of the global Carboniferous time scale and Milankovitch band cyclicity in the Donets basin, eastern Ukraine. *Geochemistry, Geophysics, Geosystems*, **11** Q0AA04, doi:10.1029/2009GC002736 (2010).

92. Somerville, I. D. Biostratigraphic zonation and correlation of Mississippian rocks in Western Europe: some case studies in the late Viséan/Serpukhovian. *Geological Journal*, **43**, 209-240 (2008).

93. Pointon, M. A., Chew, D. M., Ovtcharova, M., Sevastopulo, G. D., & Delcambre, B. High-precision U–Pb zircon CA-ID-TIMS dates from western European late Viséan bentonites. *Journal of the Geological Society*, **171**, 649-658 (2014).

95. Schmitz, M. D. & Davydov, V. I. Quantitative radiometric and biostratigraphic calibration of the Pennsylvanian–Early Permian (Cisuralian) time scale and pan-Euramerican chronostratigraphic correlation. *Geological Society of America Bulletin*, **124**, 549-577 (2012).

96. Gradstein, F. M., Ogg, J. G., Schmitz, M., & Ogg, G. (Eds.). *The Geologic Time Scale* (Elsevier, 2012).

97. Cooper R.A. & Sadler P.M. in *The Geologic Time Scale, Vol II.*(eds Gradstein, F. M., Ogg, J. G., Schmitz, M. & Ogg, G.), 489-524 (Elsevier, 2012).

98. Löfgren A. & Zhang J. Element association and morphology in some Middle Ordovician platform-equipped conodonts. *Jour. Paleontol.* **77**, 721-737 (2003).

99. Landing, E., Rushton, A. A., Fortey, R. A. & Bowring, S. A. Improved Geochronologic Accuracy and Precision for the ICS Chronostratigraphic Charts: Examples from the Late Cambrian-Early Ordovician. *Episodes*, **38**, 154-161 (2015).

100. Opdyke, M. D. & Channell, J. E. *Magnetic stratigraphy*. (Academic Press, 1996).
